# Impaired GABA synthesis, uptake and release are associated with depression-like behaviors induced by chronic mild stress

**DOI:** 10.1038/tp.2016.181

**Published:** 2016-10-04

**Authors:** K Ma, A Xu, S Cui, M-R Sun, Y-C Xue, J-H Wang

**Affiliations:** 1 Department of Pharmacology, Qingdao University, School of Pharmacy, Qingdao, China; 2 State Key Lab of Brain and Cognitive Science, Institute of Biophysics, Chinese Academy of Sciences, Beijing, China; 3 Department of Biology, College of Life Science, University of Science and Technology of China, Hefei, China; 4 Department of Biology, University of Chinese Academy of Sciences, Beijing, China

## Abstract

Major depression is a prevalent emotion disorder. Chronic stressful life in genetically susceptible individuals is presumably a major etiology that leads to neuron and synapse atrophy in the limbic system. Molecular mechanisms underlying the pathological changes remain elusive. Mice were treated by chronic unpredictable mild stress (CUMS) until they demonstrated depression-like behavior. GABA release in the medial prefrontal cortex was evaluated by cell electrophysiology and imaging. Molecular profiles related to GABA synthesis and uptake were investigated by the high-throughput sequencings of microRNAs and mRNAs as well as western blot analysis in this cortical area. In CUMS-induced depression mice, there appear the decreases in the innervation and function of GABAergic axons and in the levels of mRNAs and proteins of glutamate decarboxylase-67, vesicular GABA transporter and GABA transporter-3. miRNA-15b-5p, miRNA-144-3p, miRNA-582-5p and miRNA-879-5p that directly downregulate such mRNAs increase in this cortex. Our results suggest that chronic mild stress impairs GABA release and uptake by upregulating miRNAs and downregulating mRNAs and proteins, which may constitute the subcellular and molecular mechanisms for the lowered GABA tone in major depression.

## Introduction

Major depression, characterized as anhedonia and low self-esteem, is one of common psychiatric disorders. In terms of its pathogenesis, chronic stress to the genetically susceptible individuals leads to the dysfunctions of monoamine, brain-derived neurotrophic factor and hypothalamus–pituitary–adrenal axis,^1, 2, 3, 4, 5^ which induce the neuron atrophy in the brain reward circuits, such as the prefrontal cortex, nucleus accumbens and amygdala, in these depression patients and depression-like animals.^6, 7, 8, 9, 10, 11, 12^ These changes may be caused by a process that environmental stress evokes a series of epigenetic mechanism and gene expression.^13, 14^ As the physiological coordination between the excitatory and inhibitory neurons is critical for the neuron encoding to manage well-organized cognitions,^15, 16^ cell-specific pathology caused by molecular processes in major depressive disorder remains to be elucidated.^17, 18, 19^

The studies indicate that the decreased density of GABAergic neurons in the prefrontal cortices is associated to major depression individuals.^20, 21, 22, 23^ Inhibitory function in this cortical area appears impaired in the depression subjects.^24, 25, 26, 27, 28, 29, 30^ Current studies in depression-like mice show the decrease of cortical inhibition.^19, 31^ However, molecular mechanisms about a series of changes in epigenetics, messenger RNA (mRNA) expression and protein production remain unknown, which link chronic stress to GABAergic deficit in major depression.

We aim to reveal molecular profiles underlying the impairments specific for GABAergic neurons, such as GABA synthesis, release and uptake from depression-like mice induced by chronic unpredictable mild stress (CUMS). The capability of GABA release in the medial prefrontal cortex was evaluated by analyzing spontaneous inhibitory postsynaptic currents (sIPSCs) on glutamatergic neurons with whole-cell recording as well as GABAergic axon innervations on the glutamatergic neurons with confocal cell imaging. The capacities of GABA synthesis and uptake were merited by analyzing the genes and proteins of glutamate decarboxylase (GAD) and GABA transporters (GAT), in which the high-throughput sequencing of their mRNAs and miRNAs as well as the western blot analysis of these proteins were used for the quantification. With these analyses, we expect to figure out the pathogenic chain of stress, epigenetics, gene/protein expression and synapse dysfunction, which result in major depression.

## Materials and methods

All experiments were performed in accordance with the guideline and regulation by the Administration Office of Laboratory Animals at Beijing, China. All experimental protocols were approved by Institutional Animal Care Unit Committee in Administration Office of Laboratory Animals at Beijing, China (B10831).

### The mouse model of major depressive disorder induced by CUMS

In order to examine neuron-specific pathophysiology associated with major depressive disorders, we applied C57 Thy1-YFP/GAD1-GFP mice whose GABAergic and glutamatergic neurons were genetically labeled by green fluorescent protein (GFP) and yellow fluorescent protein (YFP), respectively.^32^ The male mice were used starting at postnatal day 21. In the first week, their body weight, locomotion, SPT and YMTs were measured to collect self-control data. The mice showing the consistent values in these measurements were separated into two groups, control and CUMS, in order to reduce the variations among them.^19^ The control mice lived without the following stresses.

On the basis of depression risk factors, such as weaknesses in cognitive function, emotional regulation, social interaction skill, circadian and stress responses,^33^ we used chronic stress to produce depression-like mice in the following principle. The mice lived in stressful environment, made efforts to challenge these conditions and experienced defeat outcomes, which drove them to feel cognitive and emotional inabilities and, in turn, to have anhedonia and low self-esteem. The procedures for CUMS mice include their adaptation, the CUMS and the behavioral tests.^19^

The stressful environments included social isolation, tilted cage, empty cage, damp sawdust cage, restraint space, white noise, strobe light and circadian disturbance.^1, 4, 34, 35, 36^ Except for the social isolation, these conditions were randomly selected to treat the mice in the manners of their separations or combinations every day. These treatments were applied ~1~14 h in durations and 1~12 h in intervals.^19^ The durations and intervals were unpredictable to these mice. This CUMS was sustained for 3 weeks until some of the mice expressed anhedonia and low self-esteem.

Whether the CUMS-treated mice in 3 weeks fell into anhedonia and low self-esteem was tested in days 29–31. The sucrose preference test (SPT) and Y-maze test (YMT) were used to assess the anhedonia, and the forced swimming test (FST) was used to estimate their self-esteem.^8, 37, 38, 39, 40^ The SPT was carried out by 1% sucrose water versus water for 4 h. The SPT value was presented as a ratio of the ingested sucrose water to the ingested sucrose water plus water. The YMT was performed by monitoring mouse staying in a special arm and other two arms for 2 min. The end of this special arm included a female mouse (named as M-arm). M-arm stay time was presented by a ratio of stay time in the M-arm to that in three arms. The FST was carried out by recording immobile time in a water cylinder (10 cm in diameter and 19 cm in depth at 25±1 °C) for 6 min. To quantify the FST, immobile time was presented. The SPT, YMT and FST were carried out in the groups of the CUMS and control. Before the SPT, the mice in the CUMS and control were deprived of food and water for 3 h to drive their intension of drinking water. In the YMT, these arms were cleaned by 70% ethanol and then by water after each test to reduce the effect of the odor on the test. The tests were carefully performed in a quiet room, with no additional stress, usinng same circadian circle for all mice and their adaptation in the test environment.^19^

An expression of depression-like behaviors was accepted if the mice in the CUMS group showed decreases in sucrose preference, M-maze stay time and latency, as well as increase in immobile time, compared with the values during their self-control period (the first week), and in the control group of the mice. The mice with the significant changes in all of three tests were defined as CUMS-induced depression-like mice or depression-like mice. The CUMS-treated mice in 3 weeks met this criterion to ~30%, implying their vulnerability to the stress. These mice were used as depression-like mice to study cell imaging, electrophysiology, protein expression and molecular profiles. It is noteworthy that certain mice without any significant change in these three tests are considered as resilience, that is, their invulnerability to stress situations, in our study. The mechanisms for stress vulnerability and invulnerability are not the current topic that will be studied in the future. As 30% of CUMS-treated mice met the depression criteria and all CUMS-treated mice did not show the change of the SPT at the end of week 1, the stressful situations in our study were thought to be mild stress.

### Brain slices and neurons

To have more healthy brain cells for whole-cell recording, we prepared cortical slices by the following procedures. The mice were anesthetized by isoflurane inhaling, and were infused by the artificial cerebrospinal fluid (ACSF) and oxygenated (95% O_2_ and 5% CO_2_) at 4 °C into their left ventricles until the bodies became cold, in which the concentrations (in mM) of the chemicals were 124 NaCl, 3 KCl, 1.2 NaH_2_PO_4_, 26 NaHCO_3_, 0.5 CaCl_2_, 4 MgSO_4_, 10 dextrose and 220 sucrose at pH 7.35. The mouse heads were immediately decapitated by guillotine and placed into this cold oxygenated ACSF with the brain isolation. The cortical slices (300 μm) in the coronal direction were cut with Vibratome in this cold oxygenated ACSF. They were held in another oxygenated ACSF (124 NaCl, 3 KCl, 1.2 NaH_2_PO_4_, 26 NaHCO_3_, 2 CaCl_2_, 2 MgSO_4_, 10 dextrose and 5 HEPES, pH 7.35) at 25 °C for 2 h. Each slice was placed into a submersion chamber (Warner RC-26G) that was perfused by the oxygenated ACSF at 31 °C for electrophysiological recording.^41, 42, 43^ Chemical reagents were from Sigma (St Louis, MO, USA).

Whole-cell recording was carried out on YFP-labeled glutamate neurons in layers III–IV of the medial prefrontal cortices under differential interference contrast-fluorescent microscope (Nikon FN-E600, Tokyo, Japan). The wavelength at 575 nm excited the fluorescence of YFP-labeled neurons. Glutamatergic neurons demonstrated the pyramidal somata and spike adaptation.^19^

### Whole-cell recording and neuronal functions

The neurons were recorded with a MultiClamp-700B amplifier under voltage clamp for their synaptic activities. Electrical signals were inputted to pClamp-10 (Axon Instrument, Foster City, CA, USA) for data acquisition and analysis. An output bandwidth of the amplifier was set at 3 kHz. The pipette solution for studying inhibitory synapses contained (mM) 130 K-gluconate, 20 KCl, 5 NaCl, 5 HEPES, 0.5 EGTA, 4 Mg-ATP, 0.5 Tris–GTP and 5 phosphocreatine.^44^ The pipette solutions were freshly made and filtered (0.1 μm). The osmolarity was 295–305 mOsmol and pipette resistance was 5–6 MΩ.

The output of GABAergic neurons was merited by recording sIPSCs on the glutamatergic neurons of the medial prefrontal cortices in the presence of 10 μM 6-Cyano-7-nitroquinoxaline-2,3-(1H,4H)-dione and 40 μM d-amino-5-phosphonovanolenic acid in the ACSF to block the ionotropic glutamatergic receptors.^45^ These slices were washed with Bicuculline (10 μM) at the end of experiments for blocking sIPSCs to examine whether synaptic responses were mediated by GABA_A_R. Pipette solution in a high concentration of chloride ions makes reversal potential to be −42 mV. sIPSCs are inward when membrane potentials are held at −65 mV.^44, 46^ sIPSC amplitudes denote the responsiveness and the density of postsynaptic receptors. sIPSC frequencies represent the probability of transmitter release from an axon terminal and the number of presynaptic axons innervated on the recorded cells.^47^ These parameters can be used to analyze presynaptic and postsynaptic mechanisms as well as to compare them with morphological data about neuronal interaction.

Data were analyzed if the recorded neurons had the resting membrane potentials negatively more than −60 mV, and action potential amplitudes more than 90 mV. The criteria for the acceptance of each experiment also included less than 5% changes in resting membrane potential, spike magnitude and input resistance throughout each recording. The series and input resistances in all neurons were monitored by injecting hyperpolarization pulses (5 mV per 50 ms), and calculated by voltage pulses versus instantaneous and steady-state currents.

### Cell imaging in the medial prefrontal cortex

The mice were anesthetized by the intraperitoneal injection of urethane (1.5 g kg^−1^), and then were perfused by 4% paraformaldehyde in 0.1 M phosphate-buffered saline into their left ventricle until their bodies were rigid. The brains were fixed in 4% paraformaldehyde for additional 24 h. The cortical tissues were sliced in a series of coronal sections (100 μm). The images for the glutamatergic and GABAergic neurons in layers III–IV were photographed under confocal microscopy with oil lens (Plan Apo VC × 60, 1.4 numerical aperture; Nikon A1R plus, Tokyo, Japan). Despite the peaks of GFP and YFP emission wavelengths being 510 and 525 nm, respectively, we separated YFP terminals and GFP-GABAergic neurons by setting the optical grating in 505–515 nm for GFP and the optical grating in 545–555 nm for YFP.

The processes of glutamatergic and GABAergic neurons were measured in each of the sections^48^ by using ImageJ (version 1.47; the National Institute of Health, Bethesda, MD, USA). In terms of the structural interaction between excitatory and inhibitory neurons, we analyzed their mutual innervations by counting the contacts of presynaptic boutons on postsynaptic neurons. These contacts were counted from the layer-by-layer of confocal cell imaging, that is, they were not the overlaps of three-dimensional imaging. YFP-labeled glutamatergic axon boutons on GFP-labeled GABAergic neurons in contacts per neuron and GFP-labeled GABAergic axon terminals on YFP-labeled glutamatergic dendrites in contacts per 100 μm length were counted.^32^ It is noteworthy that fluorescent proteins are not labeled to all neurons because of low-efficient promoters. These low densities of neuronal contacts are parallel in control and depression-like mice.

### RNA harvest from medial prefrontal cortices

Twenty-four hours after the mice were surely tested to demonstrate CUMS-induced depression-like behaviors, these depression-like mice and controls were anesthetized by using Isoflurane and decapitated by guillotine. Both sides of the medial prefrontal cortex were quickly isolated and dissected on the ice-cold glass slides. These cortical tissues were placed into the frozen vials that contained RNAlater RNA Stabilization Reagent (QIAGEN, Amtsgericht, Dusseldorf, Germany) at 4 °C and stored at −80 °C for subsequent analyses. Total RNAs from the tissue of the medial prefrontal cortex in each mouse were isolated with TRIzol Reagent (Life Technologies, Carlsbad, CA, USA) based on the manufacturer's instruction. Total RNA samples placed in the dried ice were delivered to Beijing Genomics Institute (BGI, Shenzheng, China) for sequencing analysis. RNA samples were quality-controlled by the BGI staff using Agilent 2100 Bioanalyzer (Agilent Technologies, Palo Alto, CA, USA) with RNA 6000 nano Reagents Port 1. The concentration of total RNA, the value of RNA integrity number and the ratio of 28 to 18S ribosomal RNA were measured. The samples with total RNA amount above 10 μg, the concentration above 200 ng μl^−1^, the RNA integrity number above 8 and the ratio of 28 to 18S above 1.0 were selected for the constructions of transcriptome and small RNA libraries, respectively.

### Library construction and Illumina high-throughput sequencing

mRNAs were extracted from the total RNA by oligo(dT) beads. mRNAs were randomly sheared into 200 bp fragments that were reverse-transcribed into the complementary DNA (cDNAs) by random oligonucleotides. Upon end repair, these synthesized cDNAs were purified by using the QiaQuick PCR extraction kit (Amtsgericht) and were ligated by sequencing adaptors. After the amplification with Illumina PCR Primer Cocktail within 15 cycles of PCR reaction, these cDNAs were size-selected and purified using agarose gel electrophoresis. cDNAs with sizes between 200 and 300 bp were selected for library construction.

In the meantime, miRNA sequencing library was constructed from the total RNAs, in which low molecular weight RNAs (18–30 nt) were isolated by polyacrylamide gel electrophoresis. 5′-RNA adapter was ligated to RNAs with T4 RNA ligase. The ligated RNA was size-fractionated, and 36–50 nucleotide fractions were excised. 3′-RNA adapter was subsequently ligated to the precipitated RNAs by using T4 RNA ligase. The ligated RNAs were size-fractionated and a 62–75 nucleotide fraction (small RNA+adaptor) was excised. Small RNAs ligated with adaptors were subjected to RT-PCR to produce sufficient templates for sequencing. PCR products were purified and collected by gel purification, which were ready for high-throughput sequencing.

The qualities of transcriptome and small RNA libraries were assessed by using the Agilent 2100 Bioanalyzer (Agilent Technologies). The quantity of PCR products was verified by quantitative PCR in the ABI StepOnePlus Real-Time PCR System (Foster City, CA, USA). After the quality and quantity of the prepared libraries were confirmed to be qualified, their sequencings were carried out by using Illumina HiseqTM 2500 platform (Illumina, San Diego, CA USA). The average reading length of two libraries were ~100 bp (pair-end) and 49 bp (single-end), respectively.

### Bioinformatics analysis of transcriptome

The original image data were transferred into sequence data by base calling, which were defined as raw data or raw reads. DynamicTrim Perl script implemented the in SolexaQA package was performed to control the quality of raw sequencing data based on the following criteria. (1) Remove reads with adapters. (2) Remove reads in that unknown bases were more than 10%. (3) Remove with 50% of the bases with low-quality score (PHRED score 5). After filtering, the remaining reads were called as ‘clean reads' and then mapped to mouse genome reference sequence (UCSC mm10) by using TopHat v1.0.12 (Carlsbad, CA, USA), which incorporated the Bowtie v0.11.3 software to perform the alignments. For the alignment and mapping, the maximum of allowable mismatch was set to 3 for each read. The sole reads uniquely aligned to the genes were used to calculate the gene expression level. Reads per kilo base per million reads were used for gene expression.

We screened the differentially expressed genes (DEGs) based on the NOIseq package method, which determines DEGs between two groups with biological replicates. The threshold used to identify DEGs has fold-change above 1.5 and the diverge probability above 0.8. We subsequently conducted a pathway enrichment analysis of DEGs' association with physiological or biochemical processes. In the enrichment analyses, we used hypergeometric test implemented in the tool WebGestalt (version 2) and the canonical pathways from the Kyoto Encyclopedia of Genes and Genomes database. This analysis would identify the enriched metabolic pathways or signal transduction pathways in DEGs that were compared with the whole-genome background. *P-*values from the hypergeometric tests were adjusted by the Benjamini–Hochberg method. The pathways with adjusted *P*-values less than 0.05 were considered to be significant enrichments.

### Bioinformatics analysis of miRNA expression profile

Forty-nine-nucleotide (nt) sequence tags from Hiseq sequencing were initially processed to remove adaptor sequences, low-quality reads and contaminants for the credible clean reads. To remove reads from noncoding RNA, such as ribosomal RNAs, transfer RNAs, small nuclear RNAs, small nucleolar RNAs and repeat RNA, we aligned the reads to Genbank database and Rfam database with blast or bowtie softwares. All of the high-quality clean reads ranging from 18 to 25 nt were matched to the known miRNA precursor of corresponding species in miRBase to obtain the miRNA count. The detailed criteria include the following: (1) align the tags to miRNA precursor in miRBase with no mismatch, (2) based on the first criteria, the tags align to the mature miRNA in miRBase with at least 16-nt overlap, allowing offsets. miRNAs that were satisfied with these criteria would be counted to get the expression of identified miRNAs. The remaining reads without any annotation were used to predict potential novel miRNAs and its stem loop structure using Miredp based on Friedlander *et al.*^49, 50^

We used the DESeq software algorithm that was based on negative binomial distribution and biology duplicate samples to compare the known or novel miRNA expression in control versus depression groups. The criteria for assigning significance included that *P*-value was less than 0.05 and the fold-change was larger than 1.5. Gene targets of differentially expressed miRNAs were predicted with three miRNA target prediction webs, PITA (http://genie.weizmann.ac.il/pubs/mir07/), Targetscan (http://www.targetscan.org/) and RNA22 (https://cm.jefferson.edu/rna22/). To eliminate the bias caused by single database prediction, the genes predicted by these three softwares were chosen for further analysis.

### Integrated miRNA/mRNA network analysis

In order to find the correlation between miRNAs and their target mRNAs, a series of bioinformatics analyses were performed. miRNAs usually show negative correlation with their targeted mRNAs. To identify potential miRNA-regulated target genes, the data sets of differentially expressed miRNAs and transcripts were integrated. We set the following criteria for the potential targets. The target mRNAs and miRNAs should be simultaneously and reversely changed. The target mRNAs should be predicted by miRNAs in three websites, Targetscan (http://www.targetscan.org/), PITA (http://genie.weizmann.ac.il/pubs/mir07/) and RNA22 (https://cm.jefferson.edu/rna22/), where the principle for the prediction of miRNA targets includes the matched seeds, accessible sites, free energy and conservation. Compliant miRNA target predictions were compared with those of DEGs from transcriptome sequencing to detect overlap.

### Quantitative RT-PCR for the validations of miRNA and mRNA

To validate the results from high-throughput sequencing, we used quantitative real-time RT-PCR (qRT-PCR) by the SYBR Green technique to analyze three mRNAs and four miRNAs that were involved in GABA synthesis and uptake as well as were significantly difference between control (*n*=8) and depression-like mice (*n*=8), in which the samples were used from those tissues for high-throughput sequencing. All gene primers produced the amplicons that spanned two exons. Each of them were located in the highly conserved coding areas and included all known alternatively spliced mRNA variants (Supplementary Table 1). RNAs were isolated from the tissues of the medial prefrontal cortex by using the Trizol method. cDNAs were synthesized for mRNA expression assays from total RNA in 10 μl volume with 2 μl PrimeScript RT Master Mix (TaKaRa Biotechnology, Guangzhou, China), 1 μl total RNA and 7 μl ddH_2_O. cDNAs were synthesized for miRNA expression assays from total RNA of the same sample in a 25 μl volume, including 2 μl total RNA, 2 μl reverse transcription primer (random primers for U6 small nuclear RNA and Bulge-LoopTM miRNA, RiboBio, Guangzhou, China), 5 μl RT Buffer, 0.5 μl PrimeScript II Reverse Transcriptase (200 U μl^−1^), 0.5 μl Rnase inhibitor (40 U μl^−1^), 2 μl dNTP (2.5 mM) and 13 μl ddH_2_O. qRT-PCR was done by using biosystems QuantStudio 7 Flex (Life Technologies). Each reaction was carried out in the total volume of 20 μl including 1 μl cDNA, 10 μl SYBR Premix Ex Taq II (TaKaRa Biotechnology), 0.5 μl per primer and 9 μl ddH_2_O, where the program was set to 95 °C in 5 min for pre-incubation, 40 cycles at 95 °C in 5 s and at 60 °C in 20 s for the annealing and amplification, as well as finally for the addition dissociation curve. The relative expression level of mRNAs in the tissue was normalized to internal reference gene β-actin. The relative expression level of miRNAs in the tissue was normalized to U6 small nucleolar RNA. All qRT-PCR runs were repeated in three replications. The results were calculated with the 2^−ΔΔCt^ method.

### Western blot

The tissues isolated from the medial prefrontal cortex in each sample of depression-like mice and controls were gently washed three times in ice-cold phosphate-buffered saline and placed in 1 ml of RIPA Lysate buffer with phenylmethylsulfonyl floride (Beyotime Biotechnology, Beijing, China) for their homogenizations. The homogenized tissues were removed into a new EP tube (1.5 ml), kept at 4 °C in the refrigerator for 30 min and centrifuged at 12 000 *g* per min for 15 min. Total protein concentration was measured by using protein assay based on the manufacturer's instruction in supernatant liquid. Fifty micrograms of total proteins per sample were resolved in 10% sodium dodecyl sulfate polyacrylamide gel electrophoresis. After their separations, the proteins were electrically transferred on nitrocellulose membranes. The membranes were incubated with blocking solution (1 × TBS; 0.1% Tween 20; 5% non-fat milk) at room temperature (25 °C) for 2 h, and then were incubated overnight with primary antibodies (1:2000 in dilution) of GAD-67 (ab26116, Abcam, Cambridge, UK), VGAT (A3129, ABclonal Technology, Wuhan, China), GAT-3 (AB1574, Minipore, Temecula, CA, USA) or with a primary antibody (1:1000 in dilution) of β-actin (AC004, ABclonal Technology) in 5% milk in TBST. After incubations with their corresponding secondary antibodies conjugated with peroxidase (Beyotime Biotechnology), these proteins were visualized by using the enhanced chemiluminescence ECL Plus immunoblotting detection system (Climx Science Instruments, Beijing, China). The bands corresponding to the expected sizes were selected on a computerized scanner, and the pixel density in each band was determined by this computer after the background correction for the relative quantization. The optical densities of each band relative to measured values of β-actin bands were determined using the ImageJ software.

### Dual luciferase reporter assay

Luciferase assay was performed as previously described^51^. The 3′-untranslated repeat (UTR) sequences of *GAT-3, VGAT* and *GAD1* were amplified and fused into the *Xho*I and *Not*I sites of a dual luciferase vector psiCHECK2, a generous gift from Dr Xue (Institute of Biophysics, Chinese Academy of Sciences). The site-directed mutation of the detected miRNA-targeting site of 3′-UTR fragment was constructed based on a guideline of the QuikChange Lighting Site-Directed Mutagenesis Kit (Stratagene, La Jolla, CA,USA). miRNA mimics and their negative control were synthesized by Guangzhou Rui Bo Biological Technology (Guangzhou, China). Primer sequences are listed in Supplementary Table 2. For the luciferase reporter detection, HEK293T cells were planted in RPMI media containing 10% fetal bovine serum at 5 × 10^4^ cells per well in 24-well plates. After 24 h, these cells were co-transfected with 20 ng psiCHECK2 3'-UTR wild-type or mutant reporter plasmids. In the meantime, these wells with the cell culture of 50 nM of miRNA mimic or miRNA-negative control were added by using Lipofectamine 2000 transfection reagent (Invitrogen, Carlsbad, CA, USA). The activities of firefly and Renilla luciferases were assessed after 48 h by using the Dual-Glo Luciferase Assay System (Promega, Madison, WI, USA, Cat. E2920), based on the manufacturer's protocols. Each treatment was performed in the triplicates in three independent experiments.

### Statistical analyses

The initial processing raw data of transcriptome and small RNA expression profile were performed by using NOIseq and DESeq software algorithm. Relationships between miRNA and its target prediction were assessed by Pearson's correlation coefficients. The data of behavioral tests, electrophysiology, morphology and luciferase activity are presented as mean±s.e.m. One-way analysis of variance is used to make statistical comparison in neuronal activity, morphology and molecular biology between the control and depression-like groups. The criterion for statistical significance was set at *P*<0.05.

## Results

### Chronic unpredictable mild stress induces the mice to express depression-like behaviors

**Figure 1:**
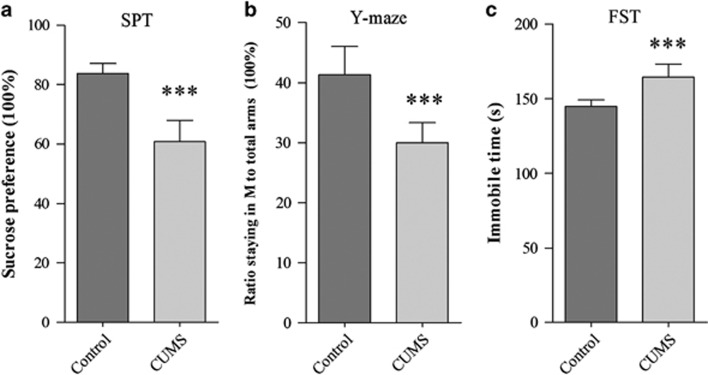
Chronic unpredictable mild stress (CUMS) leads the mice to express depression-like behaviors. Mice were subjected to the adaptation for a week, the CUMS for 3 weeks and the behavioral tests in 3 days. Behavioral tests show the significant decreases in sucrose preference (**a**) and the ratios of stay time in the M-arm to stay time in three arms (**b**) as well as an increase in immobile time (**c**) from CUMS-induced depression mice (*n*=33) and controls (*n*=30). The results are expressed as mean±s.e.m. ***P<0.001.

The mice were treated by CUMS or controls for 3 weeks. Their mood states were evaluated by SPT, YMT and FST. These procedures were described in our previous study.^19^ If CUMS-treated mice show significant changes in all of these tests, they were called CUMS-induced depression mice. As showed in Figure 1, the SPT values are 61.26±1.7% in CUMS-induced depression mice (*n*=33) and 84.47±0.85% in control mice (*n*=30, Figure 1a). The values from CUMS-induced depression mice versus control are statistically different (*P*<0.001). The ratios of stay time in the M-arm to stay time in total arms are 30.1±0.87% in CUMS-induced depression mice (*n*=33) and 42.18±1.2% in controls (*n*=30, *P*<0.001; Figure 1b). The values of immobile time in the FST are 164.4±2.1 s in CUMS-induced depression mice (*n*=33) and 144.8±1.1 s in controls (*n*=30, *P*<0.001; Figure 1c). Thus, the CUMS leads to the mice expressing depression-like behavior.

In terms of cellular and molecular mechanisms, we focused on examining the role of GABAergic neurons in the pathogenesis of major depression as these neurons were vulnerable to pathological factors^45, 52, 53, 54, 55, 56, 57, 58, 59^ and their subcellular incoordination was associated with major depression.^19^ The output ability of the GABAergic neurons was merited by analyzing sIPSC and axon innervations on their targeted glutamatergic neurons in the medial prefrontal cortex.

### The outputs of GABAergic neurons in the medial prefrontal cortex decrease from depression-like mice

**Figure 2:**
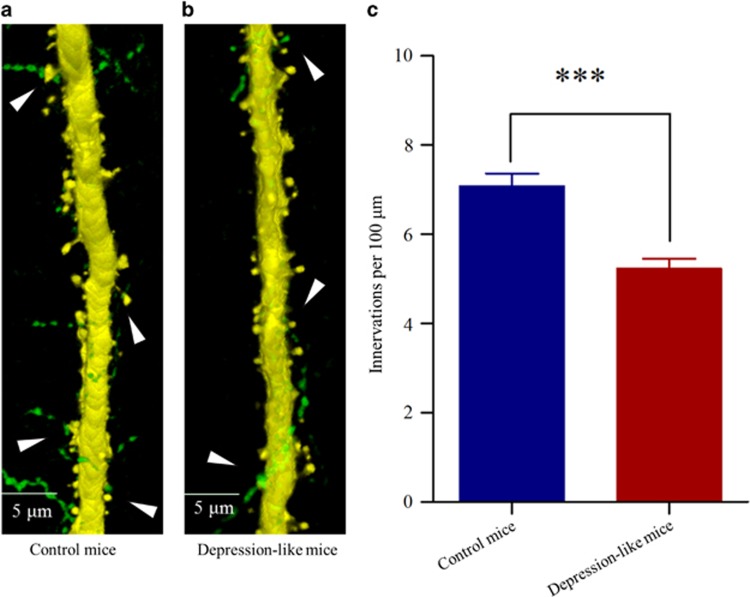
Inhibitory axon innervations on the glutamatergic neurons are downregulated in the medial prefrontal cortex (mPFC) from the depression-like mice. (**a**) The innervations of GABAergic axons (green) on the apical dendrites of glutamatergic neuron (yellow) in the mPFC from a control mouse. (**b**) The innervations of GABAergic axons (green) on the apical dendrites of glutamatergic neuron (yellow) in the mPFC from a depression-like mouse. (**c**) The comparisons of innervations per 100 μm dendrite from depression-like mice (red bar, *n*=80 apical dendrites from seven mice) and controls (blue, *n*=78 apical dendrites from six mice; ***P<0.001).

The innervations from GABAergic axons to glutamatergic neurons were counted by GFP-labeled axon terminals on YFP-labeled glutamatergic neurons. Their contacts appear decreased in CUMS-induced depression mice (Figure 2b), compared with those in controls (Figure 2a). GFP-labeled axon terminals on 100 μm of YFP-labeled apical dendrites from glutamatergic neurons are 5.23±0.23 in CUMS-induced depression mice (red bar in Figure 2c, *n*=80 apical dendrites from seven mice) and 7.08±0.29 in control (blue, *P*<0.001, *n*=78 apical dendrites from six mice). Depression-like behavior is accompanied by the decreased innervation from GABAergic axons on glutamatergic neurons in the medial prefrontal cortex.

**Figure 3:**
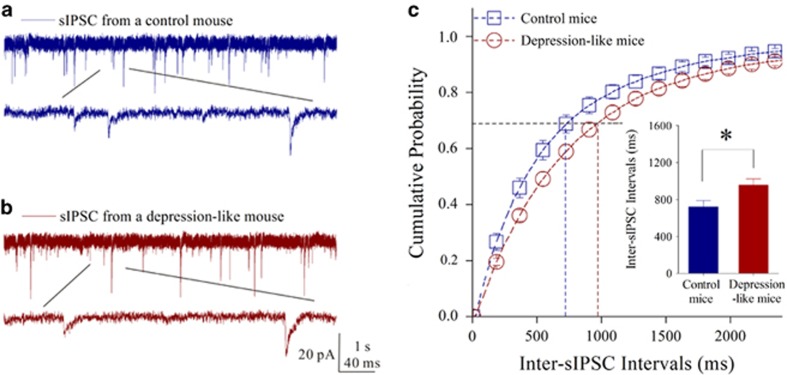
Inhibitory synaptic transmission is downregulated in the glutamatergic neurons of the medial prefrontal cortex from depression-like mice. Spontaneous inhibitory postsynaptic currenta (sIPSCs) were recorded under voltage clamp in the brain slices from control and depression-like mice in presence of 10 μM 6-Cyano-7-nitroquinoxaline-2,3-(1H,4H)-dione (CNQX) and 40 μMd-amino-5-phosphonovanolenic acid (D-AP5). (**a**) sIPSCs from a control mouse (blue traces), in which bottom trace is expanded from top trace. Calibration bars are 20 pA in vertical bar as well as 1 s (top trace) and 40 ms (bottom trace) in horizontal. (**b**) sIPSCs from a depression-like mouse (red trace), in which bottom trace is expanded from top trace. (**c**) Cumulative probability versus inter-sIPSC intervals from the depression-like mice (red symbols) and control (blue symbols). Dashed lines indicate inter-sIPSC intervals at the cumulative probability to 67% (CP_67_). Insert plot shows inter-sIPSC intervals at CP_67_ in the control (blue bar; *n*=16 cells from eight mice) and in depression-like mice (red; *n*=18 cells from 10 mice; *P<0.05).

The ability of GABA release from the presynaptic terminals of GABAergic neurons was merited by recording GABAergic sIPSCs in glutamatergic neurons. In Figure 3a, sIPSC frequency appears low in CUMS-induced depression mice, compared with that in controls. Figure 3b shows cumulative probability versus inter-sIPSC interval from two groups of mice. Inter-sIPSC-intervals at 67% cumulative probability are 955.3±68.5 ms in CUMS-induced depression mice (*n*=18 cells from 10 mice) and 720.8±65.2 ms in controls (*n*=16 cells from eight mice, *P*=0.019 in Figure 3c). Depression-like behavior is accompanied by the decreased GABA release in the medial prefrontal cortex. The decreased GABA release and GABAergic axon innervation constitute the lowered output ability of GABAergic neurons, which also strengthen the reliability of our data.

In terms of molecular mechanism underlying the attenuated output ability of GABAergic neurons, the genes and proteins related to GABA synthesis and uptake were hypothetically downregulated, which was examined by using the high-throughput sequencing of mRNA and the western blot detection of proteins related to these functional processes. The detailed integrated transcriptome and small RNA expression profiles in the medial prefrontal cortices of CUMS-induced depression mice are shown in RNA sequening of Supplementary Information.

### The genes and proteins related to GABA synthesis and transporters decrease in CUMS-depression mice

**Figure 4:**
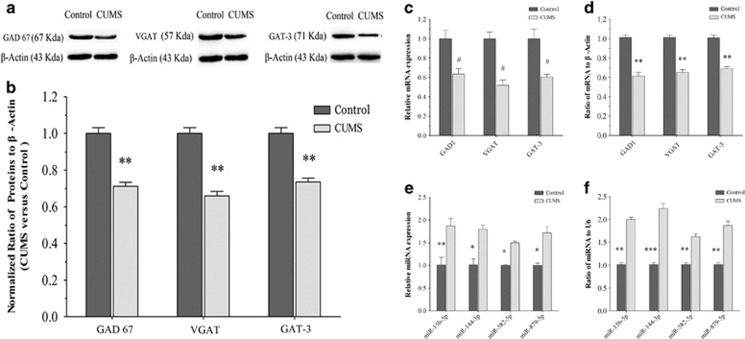
Molecular mechanisms are involved in the impaired GABA synthesis, uptake and release in the medial prefrontal cortices (mPFCs) of chronic unpredictable mild stress (CUMS)-induced depression mice. (**a**, **b**) The expression and relative quantity of proteins glutamate decarboxylase-67 (GAD-67), vesicular GAT (VGAT) and GABA transporter (GAT-3) were studied by western blot. (**a**) The expressions of GAD-67, VGAT and GAT-3 from a control mouse versus a CUMS-induced depression mouse, where internal control is performed with β-actin. (**b**) The normalized ratios of proteins (GAD-67, VGAT and GAT-3) to β-actin from control mice (dark-gray bars, *n*=8) versus CUMS-induced depression mice (light-gray bars, *n*=8). The relative ratios for control mice are normalized to be one. (**c**, **d**) The expressions and the relative quantity of messenger RNAs (mRNAs) for GAD-67, VGAT and GAT-3 were studied using high-throughput sequencing and quantitative real-time RT-PCR (qRT-PCR). (**c**) The relative values of mRNAs for GAD-67, VGAT and GAT-3 in control mice (dark-gray bars, *n*=8) and CUMS-induced depression mice (light-gray bars, *n*=8), which are analyzed using high-throughput mRNA sequencing. The relative expression levels of mRNAs in CUMS-induced depression mice are normalized to that in controls based on NOIseq package method. Diverge probability larger than 0.8 is thought as significant difference (#*P*⩾0.8). (**d**) The relative values of mRNAs for GAD-67, VGAT and GAT-3 in control mice (dark-gray bars, *n*=8) versus CUMS-induced depression mice (light-gray bars, *n*=8), which are carried out by qRT-PCR. β-actin was set as internal control. The relative values for control mice were normalized to be one. (**e**, **f**) The expressions and the relative quantity of mRNAs for miRNA-15b-5p, miRNA-144-3p, miRNA-582-5p and miRNA-879-5p were studied using high-throughput sequencing and qRT-PCR. (**e**) The relative values of miRNA-15b-5p, miRNA-144-3p, miRNA-582-5p and miRNA-879-5p from control mice (dark-gray bars, *n*=8) versus CUMS-induced depression mice (light-gray bars, *n*=8), which are analyzed using high-throughput miRNA sequencing. (**f**) The relative values of miRNA-15b-5p, miRNA-144-3p, miRNA-582-5p and miRNA-879-5p from control mice (dark-gray bars, *n*=8) versus CUMS-induced depression mice (light-gray bars, *n*=8), which are studied by qRT-PCR of miRNAs. U6 was set to be internal control. The relative values for control mice were normalized to be one.*P<0.05, **P<0.01 and ***P<0.001.

The expression of GAD (GAD-67) was used to examine GABA synthesis. The levels of GAT-3 and vesicular GAT (VGAT) were used to merit GABA uptake. Figure 4a, b shows western blot analyses about the levels of proteins of GAD-67, VGAT and GAT-3 that are harvested from the medial prefrontal cortices of CUMS-induced depression mice and controls. The densities of GAD-67, VGAT and GAT-3 bands appear lower in CUMS-induced depression mice, compared with those in controls (Figure 4a). Statistical analyses in Figure 4b demonstrate that the levels of GAD-67, VGAT and GAT-3 are significantly lower in the medial prefrontal cortices from CUMS-induced depression mice than those in control mice.

The expressions of mRNAs that translated GAD-67, VGAT and GAT-3 proteins were examined by high-throughput sequencing and quantitative RT-PCR. Figure 4c illustrates the levels of such mRNAs quantified using high-throughput sequencing, where *GAD1, VGAT* and *GAT-3* are significantly lower in the medial prefrontal cortices from CUMS-induced depression mice than those from control mice. Figure 4d illustrates the levels of such mRNAs quantified by their qRT-PCR, in which *GAD1, VGAT* and *GAT-3* are significantly lower in the medial prefrontal cortices from CUMS-induced depression mice than in those from control mice.

The consistent results from the analyses of gene and protein expressions suggest that a decreased output of prefrontal cortical GABAergic neurons may be based on the impairments of GABA uptake and synthesis. In terms of the mechanism for the downregulated mRNAs, we studied the changes of miRNAs that might downregulate the expression of mRNAs.^60, 61, 62, 63^

### miRNAs that regulate the genes for GABA synthesis and transporters rise in CUMS-depression mice

In terms of the changes of miRNAs, we paid attention to analyzing miRNA-15b-5p, miRNA-144-3p, miRNA-582-5p and miRNA-879-5p with high-throughput sequencings and qRT-PCR. The analyses of these miRNAs were based on their predicted target mRNAs that encoded GAD-67, VGAT and GAT-3 by using three miRNA targeted-gene databases (TargetScan, PITA and RNA22, please see Materials and methods). The miRNAs predicted by all of three databases were selected for our analysis. The analyzed miRNAs possess the high capacity to bind mRNAs that encode GAD-67, VGAT and GAT-3, in which the number of the binding nucleotides (especially G and C contents) is high and the free energy is low. For instance, 3′-UTRs of *GAD1* (two areas), *VGAT* (one area) and *GAT-3* (two areas) are targeted by miRNA-144-3p. 3′UTRs of *GAT-3* (one area) are targeted by miRNA-15b-5p. 3′UTRs of *VGAT* (one area) are targeted by miRNA-582-5p. 3′UTRs of *GAT-3* (one area) are targeted by miRNA-879-5p (please refer to Supplementary Table 3). It is noteworthy that there is higher conservation in these miRNA-seed regions across different vertebrate species (Supplementary Figure 1).

These miRNAs are upregulated in the medial prefrontal cortices from CUMS-induced depression mice. Figure 4e illustrates the levels of miRNAs quantified by high-throughput sequencing, in which the miRNAs to target mRNAs that encode GAD-67, VGAT and GAT-3 are significantly higher in CUMS-induced depression mice than in control. Figure 4f shows the levels of miRNAs analyzed by qRT-PCR, where the miRNAs to target *GAD1, VGAT* and *GAT-3* mRNAs are significantly higher in CUMS-induced depression mice than in controls. Moreover, we analyzed correlations between these miRNAs and their predicted target mRNAs from qRT-PCR data. There are inverse correlations between these miRNAs and their target genes predicted by bioinformatic analysis (Supplementary Figure 2).

### GAD1, GAT-3 and VGAT are the direct targets of miRNA-15b-5p, miRNA-144-3p, miRNA-582-5p and miRNA-879-5p

**Figure 5:**
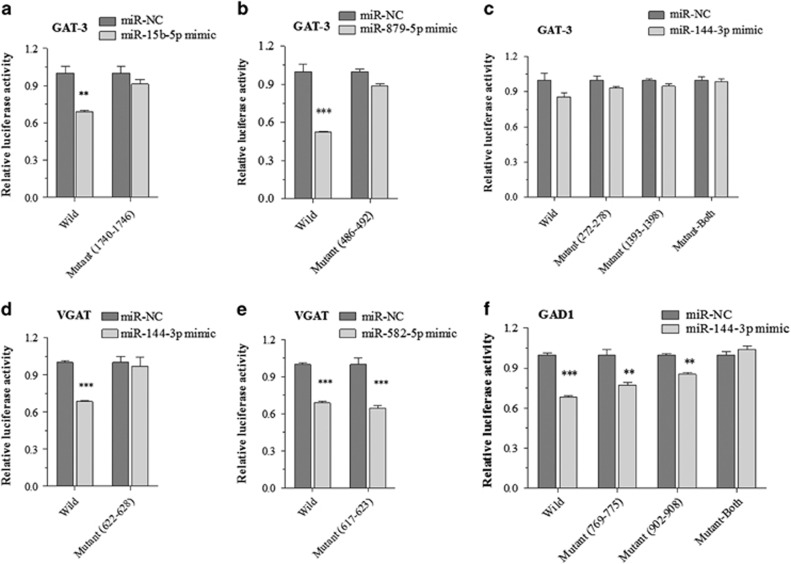
The miRNAs targeted messenger RNAs (mRNAs) for GABA synthesis and transporters are validated by Luciferase reporter assay. (**a–c**) Luciferase reporter assay is performed by the co-transfection of luciferase reporter containing wild or mutant 3′-untranslated repeat (UTR) of GABA transporter (GAT-3) mRNA with miRNA-15b-5p, miRNA-879-5p and miR-144-3p mimic or their negative control (NC) into HEK293T cells. (**d**, **e**) Luciferase reporter assay is performed by the co-transfection of luciferase reporter containing the wild or mutant 3′-UTR of vesicular GAT (VGAT) mRNA with miR-582-5p and miR-144-3p mimic or their NC into HEK293T cells. (**f**) Luciferase reporter assay is performed by the co-transfection of luciferase reporter containing the wild or mutant 3′-UTR of GAD1 mRNA with miR-144-3p mimic or their NC into HEK293T cells. Luciferase activity was determined 48 h after co-transfection. **P<0.01 and ***P<0.001.

To validate these *in silico* predictions, we analyzed the interactions between these miRNAs and their predicted target mRNAs using dual luciferase report assay. Luciferase reporter plasmids were constructed, which contained the wild-type or mutant 3′-UTRs of the predicted binding sites of the miRNAs in *GAT-3*, *VGAT* and *GAD1* mRNAs. These reporter constructs were transfected into HEK293T cells. After adding miRNA mimics or their negative controls, the relative activity of luciferase reporter for the 3′-UTR of *GAT-3* mRNA is significantly lowered by miRNA-15b-5p (Figure 5a) or miRNA-879-5p (Figure 5b) mimics. The suppressions are reversed by mutating the binding sites of miRNA-15b-5p and miRNA-879-5p (Figure 5a, b). These results suggest that *GAT-3* mRNA is a target of miR-15b-5p and miRNA-879-5p, but not a target of miRNA-144-3p (Figure 5c). Moreover, the luciferase activity of wild-type reporter for *VGAT* 3′-UTR is significantly lowered by miRNA-144-3p (Figure 5d) or miRNA-582-5p (Figure 5e). It is noteworthy that the *VGAT* suppression disappears while miRNA-144-3p binding site, but not the miRNA-582-5p-binding site, is mutated, indicating the presence of other putative binding sites in 3′-UTR of *VGAT*. The data suggest that VGAT mRNA is a target of miR-15b-5p and miRNA-879-5p. Interestingly, in the validation for miR-144-3p to target *GAD1* mRNA, the negative regulation to *GAD1* is filled by binding to two sites of *GAD1* 3′-UTR, which appears to have synergistic effect (Figure 5f). Taken together, our data suggest that *GTA-3* is directly targeted by miR-15b-5p and miRNA-879-5p, *VGAT* is targeted by miRNA-144-3p and miRNA-582-5p, as well as *GAD1* is targeted by miRNA-144-3p.

These results suggest that CUMS-induced depression may be caused by the following molecular cascades, the upregulation of miRNA-15b-5p, miRNA-144-3p, miRNA-582-5p and miRNA-879-5p, the downregulation of mRNAs that encode GAD-67, VGAT and GAT-3 as well as the impairment of GABA synthesis, reuptake and release. These correlative results in the analyses of cellular structure, function and molecules also suggest that the dysfunction of GABAergic synapses is associated with major depression induced by chronic stress.

## Discussion

**Figure 6:**
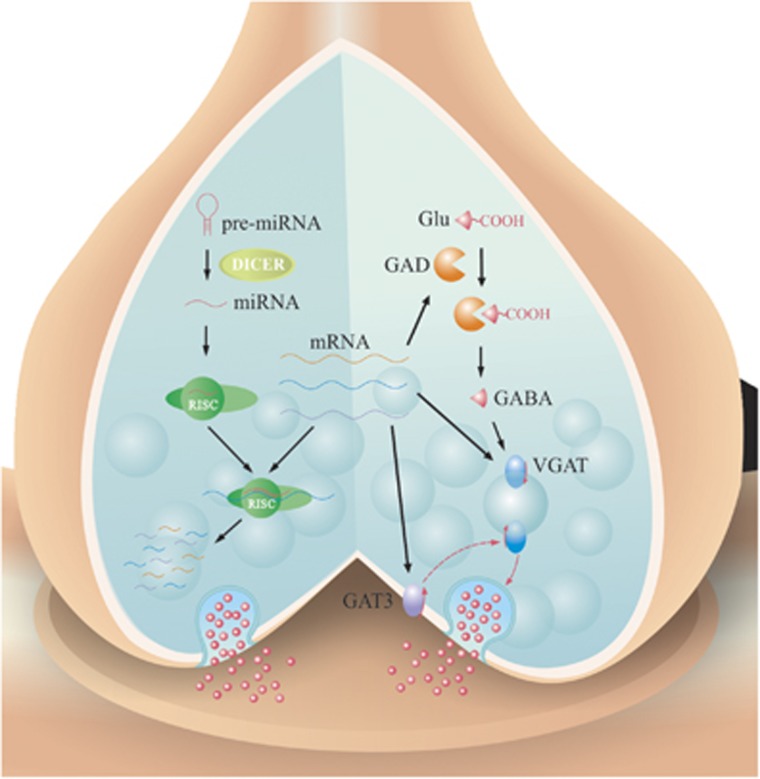
miRNAs are short (20~23 nucleotides) endogenous single-stranded RNAs that can regulate messenger RNA (mRNA) expression. The mature miRNAs and Argonaute (Ago) proteins form the RNA-induced silencing complex (RISC), a ribonucleoprotein complex that mediated post-transcriptional mRNA silencing. The complementary base pairing of miRNAs guides RISC to target their mRNAs, which are degraded, destabilized or translationally inhibited by the Ago protein. Chronic environment stress triggers a series of miRNA alteration in the medial prefrontal cortices. Subsequently, the upregulated miR-15b-5p, miRNA-879-5p, miRNA-582-5p and miRNA-144-3p directly downregulate their targeted mRNAs that encode GABA transporter (GAT)-3, vesicular GAT (VGAT) and GAD-67 proteins. Finally, these changes lead to the deterioration of GABA uptake and release in the presynaptic terminals of GABAergic neurons, which lead to the pathogenesis of major depression. GAD, glutamate decarboxylase.

In summary, GABA release and terminals decrease in the medial prefrontal cortices from CUMS-induced depression mice (Figures 2 and 3). The impairment may be caused by the decreased GABA synthesis and uptake because of the lowered expressions of GAD-67, VGAT and GAT-3 proteins and genes (Figure 4). In addition, miRNA-15b-5p, miRNA-144-3p, miRNA-582-5p and miRNA-879-5p, which downregulate mRNAs of *GAD1, VGAT* and *GAT-3*, respectively, are upregulated (Figures 4 and 5). Thus, CUMS-induced depression is likely caused by a chain reaction of the stress, the upregulated miRNA-15b-5p, miRNA-144-3p, miRNA-582-5p and miRNA-879-5p, the downregulated mRNAs that encode GAD-67, VGAT and GAT-3 as well as the impaired GABA uptake and synthesis (Figure 6).

Chronic stress to the genetically susceptible individuals presumably impairs the systems of brain-derived neurotrophic factor, monoamine and hypothalamus–pituitary–adrenal axis,^1, 2, 4, 5^ which may cause neuron atrophy in the brain reward circuits for them to suffer from major depression.^7, 10, 11, 12^ Because GABAergic neurons are vulnerable to the pathological factors,^53, 55, 56, 57, 58, 59^ their dysfunction may be the primary changes for the pathogenesis and prognosis of major depression.^19^ In this regard, the elucidation of the molecular mechanisms underlying GABAergic neuron impairment is critically important to develop the strategy and approach for the treatment of major depression.

The studies from depression subjects indicate the low GABAergic tone in the central nervous system.^24, 25, 26, 27, 28, 29, 30, 64, 65^ The enhancers of GABA_A_ receptors have been used as antidepressants; however, there is controversy in therapeutic outcomes.^56, 66, 67, 68, 69, 70^ This discrepancy may be because of the increased responsiveness or density of GABA_A_ receptors.^19^ On the other hand, GABA releases and reuptakes in presynaptic terminals' decrease by upregulating miRNAs and downregulating mRNAs/proteins related to GAD-67, VGAT and GAT-3 in CUMS-induced depression mice (Figures 2 and 5). In addition to revealing the subcellular and molecular mechanisms underlying the lowered GABA tone in major depression, our study indicates that the rescue of GAD-67, VGAT and GAT-3 productions to elevate GABA release and uptake may be the effective approach to improve depressive mood. Whether a combination of this approach with antidepressant, such as fluoxetine,^71^ have synergistic effect to treat major depression remains to be tested.

Environmental stress may trigger a series of epigenetic mechanisms and gene/protein expressions^13, 14^ via stress hormones, such as corticosterone (Supplementary Figure 3), leading to depressive mood. For instance, corticosterone induces the change of some miRNAs,^72^ which in turn affect the expressions of mRNAs and their translated proteins. It has been indicated that the stress hormones influence the function and density of GABA_A_ receptor^73, 74, 75, 76, 77^ as well as impair the reversal potential of GABA receptor channels.^78^ Our study shows that chronic stress via corticosterone upregulates the levels of miRNA-15b-5p, miRNA-144-3p, miRNA-582-5p and miRNA-879-5p as well as downregulates the expression of GAD-67, VGAT and GAT-3 genes and proteins, which impair presynaptic GABA release and uptake. These data support a hypothesis about the pathogenesis of major depression based on chronic stress, epigenetics, gene/protein expressions and neuron atrophy, in which both presynaptic and postsynaptic components in GABAergic synapses are impaired in the medial prefrontal cortex.

To address the GABA terminal deterioration and its molecular mechanism, we combined multiple approaches to strengthen our results. For instance, GABA terminal deterioration was examined by whole-cell recording to detect GABA release as well as by cell imaging to analyze GABAergic axon innervation. The alternations in the molecular mechanism of GABA release/uptake were examined by high-throughput sequencing and qRT-PCR to quantify the expressions of both mRNAs and miRNAs, and western blot analysis to quantify their encoded proteins. The correlated results from the analyses of cellular structure, function and molecules suggest that the dysfunction of GABA release and uptake is associated with major depression induced by chronic stress. In addition, using the mice with genetically YFP-labeled glutamatergic neurons and GFP-labeled GABAergic neurons in the cortices, we are able to analyze type-specific cell pathology in their subcellular compartments and mutual interactions. The stress-induced incompatibility among the subcellular compartments of the GABAergic neurons as well as the incoordination between GABAergic and glutamatergic neurons lead to imbalanced neural networks in the medial prefrontal cortex, which may be one of the bases for depressive mood.
