# Supplementary material for: Impaired GABA synthesis, uptake and release are associated with depression-like behaviors induced by chronic mild stress
Source: Transl Psychiatry. 2016 Oct 4;6(10):e910–. doi: 10.1038/tp.2016.181 (PMC5315548; doi:10.1038/tp.2016.181)
Supplement: Supplementary Information [file tp2016181x1.doc]

**Integrated transcriptome and small RNA expression profiling in the medial prefrontal cortex from CUMS-induced depression mice**

Ke Ma^1#^, Aiping Xu^2,4#^, Shan Cui^2^, Meng-Ru Sun^2^, Yuan-Chao Xue^2^ and Jin-Hui Wang^1~4^*

*1) Qingdao University, School of Pharmacy, 38 Dengzhou, Shandong China 266021; 2)*

*State Key Lab of Brain and Cognitive Science, Institute of Biophysics, Chinese Academy of Sciences, Beijing China 100101; 3) University of Chinese Academy of Sciences, Beijing China 100049; 4)College of Life Science, University of Science and Technology of China, Hefei Anhui China 230026;*

**Running title:** RNA-seq in CUMS-induced depression mice

**#** Ma, K. and Xu, A. contribute to this work equally

***Corresponding author:**

Jin-Hui Wang, Ph.D. & MD

The Institute of Biophysics, Chinese Academy of Sciences

15 Datun Road, Chaoyang District

Beijing China 100101

[jhw@sun5.ibp.ac.cn](mailto:jhw@sun5.ibp.ac.cn); 86-10-64888472

We studied molecule profile related to major depressive disorder in depression-like mice induced by chronic unpredictable mild stress (CUMS). Gene expressions were analyzed by sequencing miRNA and mRNA in the medial prefrontal cortices from CUMS-induced depression mice versus control. By these associated analyses and comparisons, we aim to figure out signal pathways in the medial prefrontal cortex related to stress-induced depression, in order to provide the guidelines for addressing the molecular mechanisms of major depressive disorder and for developing its therapeutic strategies.

We used RNA-seq to measure the transcriptome and small RNA expression profile in the same sample of the medial prefrontal cortex from each of CUMS-induced depression mice and controls with Illumina HiseqTM 2500 platform (Illumina Inc., San Diego, CA USA). The expression profile of mRNAs is presented in Table 1 if their expressions alter above 1.5 fold in depression-like mice versus controls (probability ≥ 0.8), which is the criterion to make sure gene expression alterations. 42 mRNAs are significantly downregulated in the mPFC of CUMS-induced depression mice as well as 12 mRNAs are upregulated (Figure 1). The decreased expression of mRNAs in CUMS-induced depression mice includes GAT-3, VGAT, Hap1, Gad1, Gad2, Arc, Mbp, Peg10, and so on. Based on bioinformatics of mRNA-guided protein translation (KEGG database), the downregulation of the genes and their translated proteins in Table 2 leads to the dysfunction of the following signaling pathways and processes in the medial prefrontal cortical neurons from CUMS-induced depression mice, such as GABAergic synapses, synaptic vesicle recycling and MAPK signaling and so on (Figure 2).

The level of mRNAs in the cells is affected by miRNAs, through which the bindings of miRNAs with their dicers degrade mRNAs and weaken their translations. If the downregulated mRNAs in the medial prefrontal cortex from depression mice are caused by miRNAs, their correspondent miRNAs will be upregulated. To test this hypothesis and validate our data about mRNA alternations, we analyzed the changes of miRNAs by their sequencings in CUMS-induced depression mice versus controls.

The expression profile of miRNAs is presented in Table 3 if their expressions change above 1.5 fold in all of the depression-like mice versus controls. All of the high-quality clean reads ranging from 18-25 nt were matched to the known miRNA precursor of corresponding species in miRBase to obtain the miRNA count. The detailed criteria include: 1) align the tags to miRNA precursor in miRBase with no any mismatch, 2) based on the first criteria, the tags align to the mature miRNA in miRBase with at least 16 nt overlap allowing offsets. Those miRNAs satisfied with these criteria would be counted to get the expression of identified miRNAs. Novel miRNA prediction: sRNAs which map to antisense exon, intron, or intergenic region of genome and don't map to any other RNA have a prediction of novel miRNAs. The miRNA hairpins are mostly located in intergenic regions,introns or reverse repeat sequence of coding sequence.The characteristic hairpin structure of miRNA precursor can be used to predict novel miRNA.We developed a prediction software mireap(animal/plant) or mirdeep(animal) to predict novel miRNA by exploring the secondary structure, the Dicer cleavage site and the minimum free energy of the unannotated small RNA tags which could be mapped to genome . To correct the biased results from low expression, we discarded miRNAs with read counts less than 5 in the differential expression analysis. These upregulated miRNAs include certain known miRNAs (mmu-miR-148b-5p, mmu-miR-879-5p, mmu-miR-144-3p, mmu-miR-540-5p, mmu-miR-582-5p, mmu-miR-15b-5p, mmu-miR-210-5p, mmu-miR-871-3p, mmu-miR-3103-5p, mmu-miR-16-1-3p, mmu-miR-470-5p, mmu-miR-190b-5p, mmu-miR-384-5p and mmu-miR-490-5p), as well as some novel miRNAs (novel_mir_46, novel_mir_214 and novel_mir_213) with their stem loop structures by Miredp (Figure 3). To identify potential miRNA-regulated target genes, the datasets of differentially expressed miRNAs and transcripts were integrated. We set the following criteria for the potential targets. The target mRNAs and miRNAs should be simultaneously and reversely changed. The target mRNAs should be predicted by known and novelk miRNAs in two softwares of TargetScan, PicTar and RNA22. The compliant miRNA target predictions were compared with those of DEGs from transcriptome sequencing to detect overlap. Table 4 shows the alternated miRNAs and their predicted-target mRNAs.

We have analyzed the quantitative changes of mRNAs and miRNAs in the medial prefrontal cortices from CUMS-induced depression mice versus control mice by sequencing both mRNAs and miRNAs to quantify their expression levels. mRNAs are downregulated that encode the neural processes, such as GABAergic synapse, synaptic vesicle cycle and axon grow. By reading Table 1-4 and Figure 1-2, we find that the upregulated miRNA levels and decreased mRNA expression levels of the miRNA-target genes GAD-67, VGAT and GAT-3 in the mouse depression-like model. Furthermore, previous studies have pointed out the altered dynamics of neural transmission at the synaptic end of maladapted GABAergic system and ascribed as common denominator of MDD.

Taken together, we choese GABA synthesis, uptake and release associated mRNAs (GAD 67, VGAT, GAT-3) and their associated microRNA ( miR-15b-5p, miR-144-3p, miR-582-5p and miR-879-5p) to further investigate the molecular epigenetics as underlying cause of that dysfunctionality in a genetically aidedmurine system.

**Reference**

1. Friedlander MR, Lizano E, Houben AJ, Bezdan D, Banez-Coronel M, Kudla G*, et al*. Evidence for the biogenesis of more than 1,000 novel human microRNAs. *Genome biology* 2014; **15**(4)**:** R57.

2. Friedlander MR, Mackowiak SD, Li N, Chen W, Rajewsky N. miRDeep2 accurately identifies known and hundreds of novel microRNA genes in seven animal clades. *Nucleic acids research* 2012; **40**(1)**:** 37-52.

**Figure and figure legends**

**Figure 1:** Scatter plots of all expressed genes in control Vs CUMS-induced depression like mice. A: X-axis and Y axis present log2 value of gene expression, blue means down-regulation gene, orange means upregulation gene, brown means non-regulation gene. B: Ech column represents an experimental subject, each row represents a gene. Expression differences are shown in different colors. Red means up regulation and green means down regulation.

**Figure 2:** Scatter plot of KEGG pathway enriment statistics. Rich Factor is the ratio of differentially expressed gene numbers annoted in this pathway term to all gene numbers annoted in this pathway term. Greater richFator means greater intensiveness. Qvalue is corrected pvalue ranging from 0~1, and its less value means greater intensiveness.

**Figure 3**: Stem loop structure and predicted mature sequence. A: The remained reads without any annotation were used to predict potential novel miRNAs and its stem loop structure by Miredp. B: The stem loop sequence information of predict potential novel miRNAs. Red indicates the predicted novel miRNA mature nucleotide sequence.

**Table 1. mRNAs with differential expression over 1.5 folds** and their characteristics

| Symbol | GeneID | GeneLength | Up-Down (CUMS/Control) | Fold change (CUMS/Control) | Probability | Chromosomal Map | Description |
| --- | --- | --- | --- | --- | --- | --- | --- |
| **Gad1** | **14415** | **3231** | **Down** | **0.659166** | **0.808415** | **2 D** | **glutamate decarboxylase 1** |
| **GAT-3** | **243616** | **4066** | **Down** | **0.632232** | **0.819693** | **6 E3** | **neurotransmitter transporter, GABA** |
| **VGAT** | **22348** | **2805** | **Down** | **0.582285** | **0.838340** | **2** | **GABA vesicular transporter** |
| Mbp | 17196 | 2060.69 | Down | 0.663277 | 0.808471 | 18 E2-E4 | myelin basic protein |
| Htra1 | 56213 | 2051 | Down | 0.642924 | 0.814490 | 7 F3 | HtrA serine peptidase 1 |
| Hap1 | 15114 | 3608.13 | Down | 0.630221 | 0.822365 | 11 D | huntingtin-associated protein 1 |
| Mobp | 17433 | 2729.19 | Down | 0.626003 | 0.821802 | 9 F4 | myelin-associated oligodendrocytic basic protein |
| Cldn11 | 18417 | 1872 | Down | 0.625964 | 0.823124 | 3 A3 | claudin 11 |
| Zcchc12 | 72693 | 2206 | Down | 0.624823 | 0.819159 | X A3.3 | zinc finger, CCHC domain containing 12 |
| Sparc | 20692 | 2299 | Down | 0.617425 | 0.829846 | 11 B1 | secreted acidic cysteine rich glycoprotein |
| Nrsn2 | 228777 | 1437 | Down | 0.614833 | 0.824418 | 2 G3 | neurensin 2 |
| Dusp1 | 19252 | 1952 | Down | 0.605349 | 0.815474 | 17 A2-C | dual specificity phosphatase 1 |
| Gng4 | 14706 | 2976 | Down | 0.597044 | 0.819327 | 13 A1 | guanine nucleotide binding protein |
| Npas4 | 225872 | 3292 | Down | 0.593762 | 0.810412 | 19 A | neuronal PAS domain protein 4 |
| Cryab | 12955 | 1185 | Down | 0.588849 | 0.833305 | 9 A5.3 | crystallin, alpha B |
| Gpx3 | 14778 | 1517 | Down | 0.557776 | 0.800456 | 11 | glutathione peroxidase 3 |
| Gad2 | 14417 | 5625 | Down | 0.545072 | 0.843796 | 2 A3 | glutamic acid decarboxylase 2 |
| Calb2 | 12308 | 1462 | Down | 0.529868 | 0.844246 | 8 E1 | calbindin 2 |
| Cacna2d2 | 56808 | 5515.08 | Down | 0.492444 | 0.851783 | 9 F1 | calcium channel, voltage-dependent, alpha 2/delta subunit 2 |
| Fos | 14281 | 2107 | Down | 0.479789 | 0.872764 | 12 D2 | FBJ osteosarcoma oncogene |
| Arc | 11838 | 3058.3 | Down | 0.478323 | 0.882017 | 15 D3 | activity regulated cytoskeletal-associated protein |
| Agxt2l1 | 71760 | 2704 | Down | 0.462973 | 0.813787 | 3 G3 | alanine-glyoxylate aminotransferase 2-like 1 |
| Ngb | 64242 | 1630 | Down | 0.44876 | 0.828355 | 12 | neuroglobin |
| Cyr61 | 16007 | 2028 | Down | 0.42837 | 0.834683 | 3 H2 | cysteine rich protein 61 |
| Itih3 | 16426 | 2832 | Down | 0.409737 | 0.868883 | 14 | inter-alpha trypsin inhibitor, heavy chain 3 |
| Zic1 | 22771 | 3350 | Down | 0.394804 | 0.869614 | 9 E3.3 | zinc finger protein of the cerebellum 1 |
| Peg10 | 170676 | 6677 | Down | 0.358726 | 0.854792 | 6 A1 | paternally expressed 10 |
| Nts | 67405 | 1237 | Down | 0.320956 | 0.864130 | 10 D1 | neurotensin |
| Th | 21823 | 1757 | Down | 0.313625 | 0.847789 | 7 F5 | tyrosine hydroxylase |
| Doc2g | 60425 | 1481 | Down | 0.312355 | 0.881792 | 19 A | double C2, gamma |
| Ecel1 | 13599 | 2728 | Down | 0.308565 | 0.893323 | 1 D | endothelin converting enzyme-like 1 |
| Baiap3 | 545192 | 4645 | Down | 0.30433 | 0.901817 | 17 A3.3 | BAI1-associated protein 3 |
| Ngfr | 18053 | 3409 | Down | 0.293137 | 0.807993 | 11 D | nerve growth factor receptor |
| Agt | 11606 | 1856 | Down | 0.282564 | 0.884689 | 8 E2 | angiotensinogen |
| Dlk1 | 13386 | 4355.26 | Down | 0.253205 | 0.883226 | 12 E-F1 | delta-like 1 homolog |
| Prkcd | 18753 | 2790 | Down | 0.249637 | 0.906626 | 14 B | protein kinase C, delta |
| A230065H16Rik | 380787 | 657 | Down | 0.219697 | 0.885308 | 12 F1 | RIKEN cDNA A230065H16 gene |
| Magel2 | 27385 | 4662 | Down | 0.200528 | 0.804534 | 7 C | melanoma antigen, family L, 2 |
| Igsf1 | 209268 | 3119.72 | Down | 0.185684 | 0.877995 | X A5 | immunoglobulin superfamily, member 1 |
| Tmem254c | 1E+08 | 1449 | Down | 0.18251 | 0.850405 | 14 A3 | transmembrane protein 254c |
| S100a5 | 20199 | 432 | Down | 0.003992 | 0.834936 | 3 F1-F2 | S100 calcium binding protein A5 |
| Cers1 | 93898 | 2741 | Down | 0.002134 | 0.917398 | 8 B3.3 | ceramide synthase 1 |
| Gm20878 | 1E+08 | 377 | Up | 257.5 | 0.839071 | 4 | predicted gene, 21586 |
| Gm21586 | 1.01E+08 | 377 | Up | 257.5 | 0.839071 | 4 | predicted gene, 20878 |
| S100a8 | 20201 | 392 | Up | 11.92 | 0.872427 | 3 F1-F2 | S100 calcium binding protein A8 |
| Tmem254b | 1E+08 | 1447 | Up | 2.524064 | 0.835583 | 14 A3 | transmembrane protein 254b |
| Gm129 | 229599 | 1395 | Up | 2.097105 | 0.849055 | 3 F2.1 | predicted gene 129 |
| Rs5-8s1 | 790956 | 157 | Up | 2.045579 | 0.847030 | 17 | 5.8S ribosomal RNA |
| Hba-a2 | 110257 | 587 | Up | 1.993966 | 0.875267 | 11 | hemoglobin alpha, adult chain 2 |
| Beta-s | 1.01E+08 | 639 | Up | 1.746662 | 0.846243 | 7 | hemoglobin subunit beta-1-like |
| Hba-a1 | 15122 | 569 | Up | 1.624516 | 0.830999 | 11 A4 | hemoglobin alpha, adult chain 1 |
| Hbb-b1 | 15129 | 626 | Up | 1.622396 | 0.819468 | 7 E3 | hemoglobin, beta adult major chain |
| Nr1d1 | 217166 | 2627 | Up | 1.585232 | 0.818652 | 11 D | nuclear receptor subfamily 1, group D, member 1 |
| Flot2 | 14252 | 2643.34 | Up | 1.529039 | 0.801131 | 11 B5 | flotillin 2 |

**Table 2: Signaling pathways identified by KEGG function analysis based on DEGs data**

| Pathway | DEGs with pathway annotation (45) | All genes with pathway annotation (16857) | Contributing Genes | Rich Factor | P-value^a^ | Pathway ID |
| --- | --- | --- | --- | --- | --- | --- |
| **[GABAergic synapse](../../../../C:%5CUsers%5CAdministrator%5CDesktop%5CTranscriptome%5CmRNA%20analysis%5CKEGG%20pathways.xls#RANGE!gene2)** | **6 (13.33%)** | **118 (0.7%)** | **Slc6a11(GAT3), Slc32a1(VGAT), Gad1, Hap1,Gad2, Gng4** | **0.050847** | **0.000001** | **ko04727** |
| [Neurotrophin signaling pathway](../../../../C:%5CUsers%5CAdministrator%5CDesktop%5CTranscriptome%5CmRNA%20analysis%5CKEGG%20pathways.xls#RANGE!gene7) | 4 (8.89%) | 242 (1.44%) | Magel2, Prkcd, Ngfr,Peg10 | 0.016529 | 0.003892 | ko04722 |
| MAPK signaling pathway | 5 (11.11%) | 403 (2.39%) | Dusp1, Itih3, Cacna2d2, Fos, Peg10 | 0.012407 | 0.004241 | ko04010 |
| [Dopaminergic synapse](../../../../C:%5CUsers%5CAdministrator%5CDesktop%5CTranscriptome%5CmRNA%20analysis%5CKEGG%20pathways.xls#RANGE!gene16) | 3 (6.67%) | 193 (1.14%) | Fos, Gng4,Th | 0.015544 | 0.014738 | ko04728 |
| Amphetamine addiction | 3 (6.67%) | 126 (0.75%) | Arc,Fos,Th | 0.023810 | 0.004602 | ko05031 |
| [Chemokine signaling pathway](../../../../C:%5CUsers%5CAdministrator%5CDesktop%5CTranscriptome%5CmRNA%20analysis%5CTranscriptome%5CmRNA%20analysis%5CTranscriptome%5CFC_1.5%5CFC_1.5%5CPathway%5CCtl-VS-CUMS_map%5Cmap04062.html) | 4 (8.89%) | 281 (1.67%) | Gm21586,Prkcd,Gm20878,Gng4 | 0.014235 | 0.006585 | ko04062 |
| [Cytokine-cytokine receptor interaction](../../../../C:%5CUsers%5CAdministrator%5CDesktop%5CTranscriptome%5CmRNA%20analysis%5CTranscriptome%5CmRNA%20analysis%5CTranscriptome%5CFC_1.5%5CFC_1.5%5CPathway%5CCtl-VS-CUMS_map%5Cmap04060.html) | 4 (8.89%) | 348 (2.06%) | Gm20878,Gm21586,Ngfr,Peg10 | 0.011494 | 0.013673 | ko04060 |
| [Synaptic vesicle cycle](../../../../C:%5CUsers%5CAdministrator%5CDesktop%5CTranscriptome%5CmRNA%20analysis%5CTranscriptome%5CmRNA%20analysis%5CTranscriptome%5CFC_1.5%5CFC_1.5%5CPathway%5CCtl-VS-CUMS_map%5Cmap04721.html) | 2 (4.44%) | 119 (0.71%) | Doc2g,Slc32a1 | 0.016807 | 0.040170 | ko04721 |
| [Renin-angiotensin system](../../../../C:%5CUsers%5CAdministrator%5CDesktop%5CTranscriptome%5CmRNA%20analysis%5CTranscriptome%5CmRNA%20analysis%5CTranscriptome%5CFC_1.5%5CFC_1.5%5CPathway%5CCtl-VS-CUMS_map%5Cmap04614.html) | 2 (4.44%) | 26 (0.15%) | Agt,Peg10 | 0.076923 | 0.002174 | ko04614 |
| [Morphine addiction](../../../../C:%5CUsers%5CAdministrator%5CDesktop%5CTranscriptome%5CmRNA%20analysis%5CTranscriptome%5CmRNA%20analysis%5CTranscriptome%5CFC_1.5%5CFC_1.5%5CPathway%5CCtl-VS-CUMS_map%5Cmap05032.html) | 2 (4.44%) | 113 (0.67%) | Gng4,Slc32a1 | 0.017699 | 0.036569 | ko05032 |
| [Chagas disease (American trypanosomiasis)](../../../../C:%5CUsers%5CAdministrator%5CDesktop%5CTranscriptome%5CmRNA%20analysis%5CKEGG%20pathways.xls#RANGE!gene20) | 2 (4.44%) | 139 (0.82%) | Fos,Peg10 | 0.014388 | 0.053078 | ko05142 |
| [African trypanosomiasis](../../../../C:%5CUsers%5CAdministrator%5CDesktop%5CTranscriptome%5CmRNA%20analysis%5CKEGG%20pathways.xls#RANGE!gene1) | 5 (11.11%) | 60 (0.36%) | Beta-s,Hbb-b1,Hba-a2,Hba-a1,Peg10 | 0.083333 | 0.000001 | ko05143 |
| [Malaria](../../../../C:%5CUsers%5CAdministrator%5CDesktop%5CTranscriptome%5CmRNA%20analysis%5CTranscriptome%5CmRNA%20analysis%5CTranscriptome%5CFC_1.5%5CFC_1.5%5CPathway%5CCtl-VS-CUMS_map%5Cmap05144.html) | 4 (8.89%) | 80 (0.47%) | Beta-s,Hbb-b1,Hba-a2,Hba-a1 | 0.050000 | 0.000060 | ko05144 |
| [Taurine and hypotaurine metabolism](../../../../C:%5CUsers%5CAdministrator%5CDesktop%5CTranscriptome%5CmRNA%20analysis%5CTranscriptome%5CmRNA%20analysis%5CTranscriptome%5CFC_1.5%5CFC_1.5%5CPathway%5CCtl-VS-CUMS_map%5Cmap00430.html) | 2 (4.44%) | 14 (0.08%) | Gad2,Gad1 | 0.142857 | 0.000621 | ko00430 |
| [Type I diabetes mellitus](../../../../C:%5CUsers%5CAdministrator%5CDesktop%5CTranscriptome%5CmRNA%20analysis%5CTranscriptome%5CmRNA%20analysis%5CTranscriptome%5CFC_1.5%5CFC_1.5%5CPathway%5CCtl-VS-CUMS_map%5Cmap04940.html) | 3 (6.67%) | 86 (0.51%) | Gad2,Gad1,Peg10 | 0.034884 | 0.001558 | ko04940 |
| [Alanine, aspartate and glutamate metabolism](../../../../C:%5CUsers%5CAdministrator%5CDesktop%5CTranscriptome%5CmRNA%20analysis%5CTranscriptome%5CmRNA%20analysis%5CTranscriptome%5CFC_1.5%5CFC_1.5%5CPathway%5CCtl-VS-CUMS_map%5Cmap00250.html) | 2 (4.44%) | 41 (0.24%) | Gad2,Gad1 | 0.048780 | 0.005348 | ko00250 |
| [beta-Alanine metabolism](../../../../C:%5CUsers%5CAdministrator%5CDesktop%5CTranscriptome%5CmRNA%20analysis%5CTranscriptome%5CmRNA%20analysis%5CTranscriptome%5CFC_1.5%5CFC_1.5%5CPathway%5CCtl-VS-CUMS_map%5Cmap00410.html) | 2 (4.44%) | 46 (0.27%) | Gad2,Gad1 | 0.043478 | 0.006694 | ko00410 |
| [Intestinal immune network for IgA production](../../../../C:%5CUsers%5CAdministrator%5CDesktop%5CTranscriptome%5CmRNA%20analysis%5CTranscriptome%5CmRNA%20analysis%5CTranscriptome%5CFC_1.5%5CFC_1.5%5CPathway%5CCtl-VS-CUMS_map%5Cmap04672.html) | 2 (4.44%) | 59 (0.35%) | Gm20878,Gm21586 | 0.033898 | 0.010825 | ko04672 |
| [Taurine and hypotaurine metabolism](../../../../C:%5CUsers%5CAdministrator%5CDesktop%5CTranscriptome%5CmRNA%20analysis%5CKEGG%20pathways.xls#RANGE!gene4) | 2 (4.44%) | 14 (0.08%) | Gad2,Gad1 | 0.142857 | 0.000621 | ko00430 |
| [Butanoate metabolism](../../../../C:%5CUsers%5CAdministrator%5CDesktop%5CTranscriptome%5CmRNA%20analysis%5CKEGG%20pathways.xls#RANGE!gene13) | 2 (4.44%) | 53 (0.31%) | Gad2,Gad1 | 0.037736 | 0.008807 | ko00650 |

^a^ *P-*values from the hypergeometric tests were adjusted by Benjamini-Hochberg method

**Table 3. miRNAs with quantitative change over 1.5 folds** and their characteristics

| Known miRNA | Accession no. | Up-Down (CUMS/Control) | Fold change (CUMS/Control) | p-value | Chromosomal location (mouse) | Seed sequence |
| --- | --- | --- | --- | --- | --- | --- |
| **mmu-miR-879-5p** | **MIMAT0004842** | **Up** | **2.004651** | **0.047942** | **chr5: 9375704-9375779 [+]** | **5'-gaggcuu-3'** |
| **mmu-miR-144-3p** | **MIMAT0000156** | **Up** | **1.786177** | **0.005340** | **chr11: 78073005-78073070 [+]** | **5'-acaguau-3'** |
| **mmu-miR-582-5p** | **MIMAT0005291** | **Up** | **1.718269** | **0.001605** | **chr13: 109324744-109324824 [+]** | **5'-uacaguu-3'** |
| **mmu-miR-15b-5p** | **MIMAT0000124** | **Up** | **1.870152** | **0.007213** | **chr3: 69009772-69009835 [+]** | **5'-agcagca-3'** |
| mmu-miR-540-5p | MIMAT0004786 | Up | 1.756222 | 0.000003 | chr12: 109586080-109586146 [+] | 5'-aaggguc-3' |
| mmu-miR-148b-5p | MIMAT0017036 | Up | 1.513934 | 0.000393 | chr15: 103285125-103285221 [+] | 5'-aaguucu-3' |
| mmu-miR-7240-5p | MIMAT0028448 | Up | 1.642455 | 0.010544 | chr8: 70798135-70798192 [+] | 5'-uggagag-3' |
| mmu-miR-210-5p | MIMAT0017052 | Up | 1.557688 | 0.000017 | chr7: 141221384-141221493 [-] | 5'-gccacug-3' |
| mmu-miR-871-3p | MIMAT0017265 | Up | 1.563748 | 0.000011 | chrX: 66810428-66810504 [-] | 5'-gacuggc-3' |
| mmu-miR-3103-5p | MIMAT0014937 | Up | 1.739941 | 0.000865 | chr7: 128288369-128288435 [-] | 5'-gagggag-3' |
| mmu-miR-16-1-3p | MIMAT0004625 | Up | 1.588207 | 0.001608 | chr14: 61631880-61631972 [-] | 5'-caguauu-3' |
| mmu-let-7a-1-3p | MIMAT0004620 | Up | 1.841878 | 0.022997 | chr13: 48538179-48538272 [-] | 5'-uauacaa-3' |
| mmu-let-7c-2-3p | MIMAT0005439 | Up | 1.841878 | 0.002300 | chr15: 85706603-85706697 [+] | 5'-uauacaa-3' |
| mmu-miR-470-5p | MIMAT0002111 | Up | 2.152779 | 0.033824 | chrX: 66813951-66814025 [-] | 5'-ucuugga-3' |
| mmu-miR-218-2-3p | MIMAT0005444 | Up | 2.376733 | 0.016864 | chr11: 35616816-35616925 [+] | 5'-augguuc-3' |
| mmu-miR-190b-5p | MIMAT0004852 | Up | 1.765445 | 0.000257 | chr3: 90070020-90070099 [+] | 5'-gauaugu-3' |
| mmu-miR-6240 | MIMAT0024861 | Up | 2.849409 | 0.020825 | chr5: 114851948-114852064 [+] | 5'-caaagca-3' |
| mmu-miR-3083-3p | MIMAT0014875 | Up | 2.675919 | 0.004859 | chr17: 26948056-26948119 [-] | 5'-ccgaaac-3' |
| mmu-miR-3072-3p | MIMAT0014853 | Up | 6.083997 | 0.020561 | chr12: 109747878-109747960 [+] | 5'-gcccccu-3' |
| mmu-miR-384-5p | MIMAT0004745 | Down | 0.574941 | 0.010486 | chrX: 105344282-105344369 [-] | 5'-guaaaca-3' |
| mmu-miR-1264-3p | MIMAT0014803 | Down | 0.528572 | 0.000058 | chrX: 147010601-147010686 [+] | 5'-aaaucuu-3' |
| mmu-miR-217-5p | MIMAT0000679 | Down | 0.217350 | 0.001059 | chr11: 28763728-28763835 [+] | 5'-acugcau-3' |
| mmu-miR-490-5p | MIMAT0017261 | Down | 0.671760 | 0.000251 | chr6: 36421742-36421825 [+] | 5'-cauggau-3' |
| novel_mir_46 |  | Up | 2.219962 | 0.026944 | chr2:181459599-181459621 | 5'-agcgggc-3' |
| novel_mir_214 | Only in CUMS | Up | Inf | 0.026173 | chr4:41858835-41858855 | 5'-actgcct-3' |
| novel_mir_213 | Only in CUMS | Up | Inf | 0.043573 | chr3:121770702-121770723 | 5'-ctgagcc-3' |
| novel_mir_10 | Only in control | Down | Inf | 0.000021 | chr11:105913296-105913317 | 5'-ttgaaag-3' |
| novel_mir_39 | Only in control | Down | Inf | 0.004927 | chr1:25228756-25228777 | 5'-gctggac-3' |
| novel_mir_128 | Only in control | Down | Inf | 0.008822 | chr17:26948060-26948081 | 5'-aggctgg-3' |
| novel_mir_54 | Only in control | Down | Inf | 0.035147 | chr4:41858835-41858857 | 5'-actgcct -3' |

**Table 4. The changed miRNAs predict target mRNAs**

| miRNAs | The predicted target mRNAs that match DEGs in transcriptome * |
| --- | --- |
| **mmu-miR-879-5p↑** | **Slc6a11(GAT-3)↓** |
| **mmu-miR-144-3p↑** | **Slc6a11(GAT-3)↓,GAD1↓, Slc32a1 (VGAT)↓** |
| **mmu-miR-582-5p↑** | **Slc32a1 (VGAT)↓** |
| **mmu-miR-15b-5p↑** | **Slc6a11(GAT-3)↓, Gad2↓, Ngfr↓, Gng4↓, Peg10↓** |
| mmu-miR-210-5p↑ | Mobp↓ |
| mmu-miR-871-3p↑ | Igsf1↓ |
| mmu-miR-470-5p ↑ | Fos↓, Peg10↓ |
| novel_mir_46 ↑ | Baiap3↓, Cacna2d2↓, Peg10↓ |
| novel_mir_214 ↑ | Dlk1↓, Peg10↓ |
| novel_mir_213↑ | Dusp1↓ |
| novel_mir_128↓ | Flot2↑ |

* Note: The target mRNAs should be predicted by more than two softwares of PITA, Targetscan and RNA22, and then overlapped to DEGs in transcriptome sequencing.


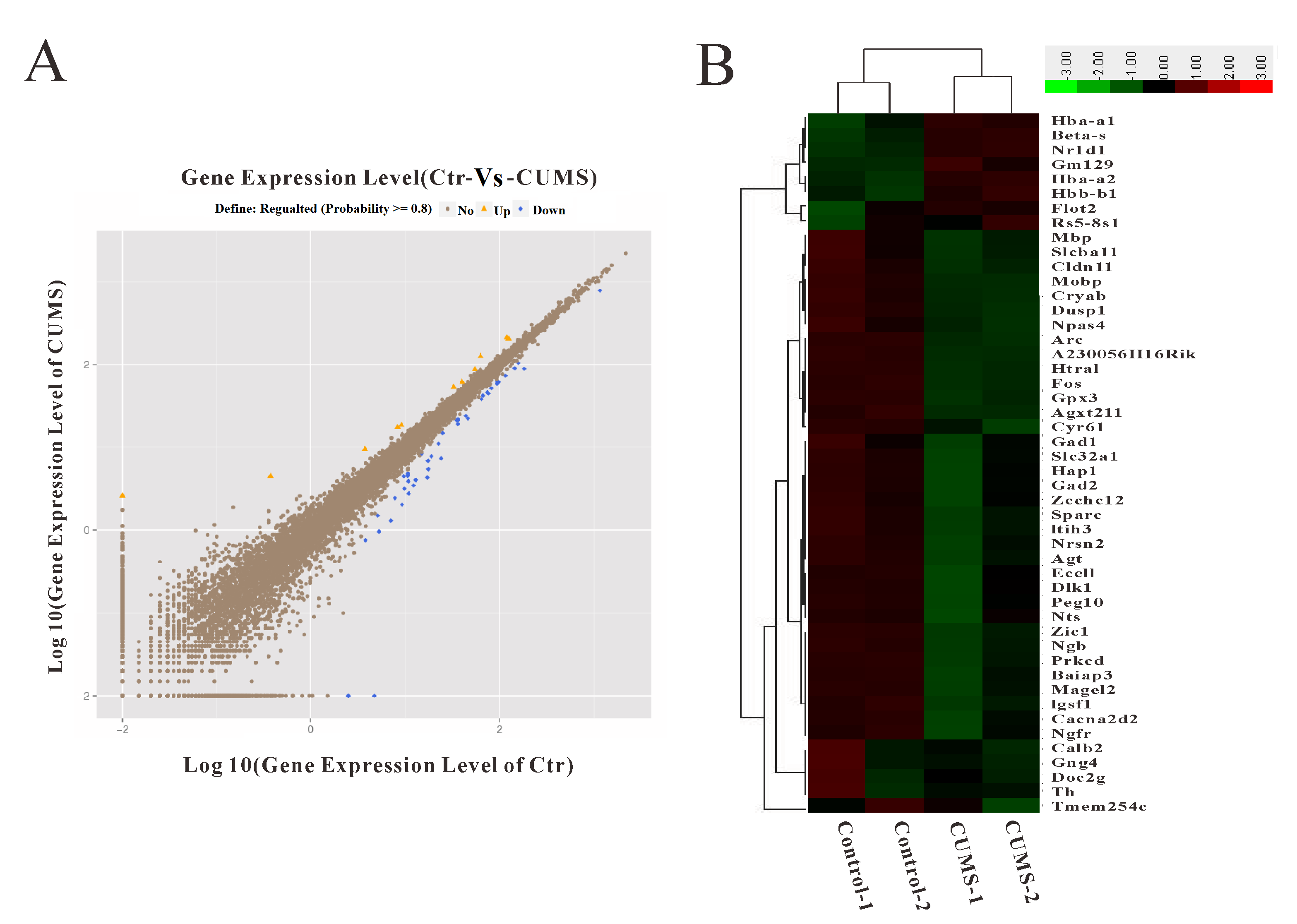


**Figure 1**


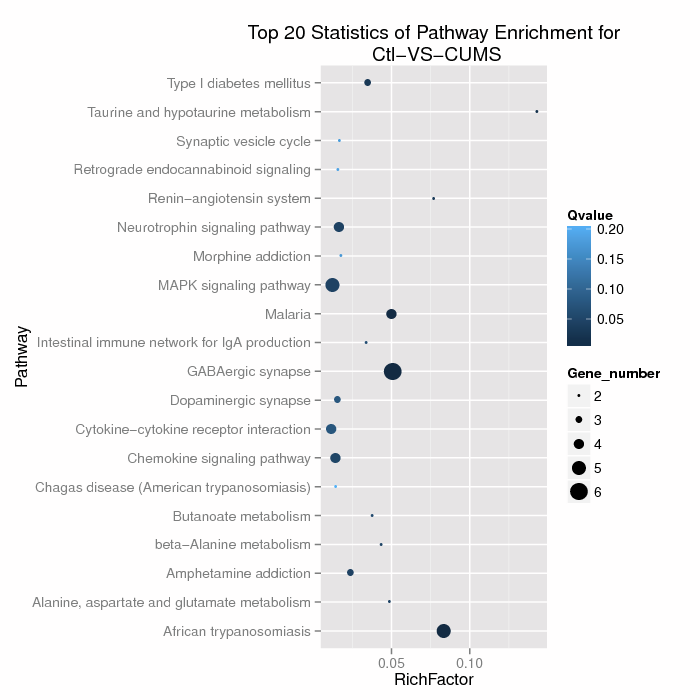


**Figure 2**


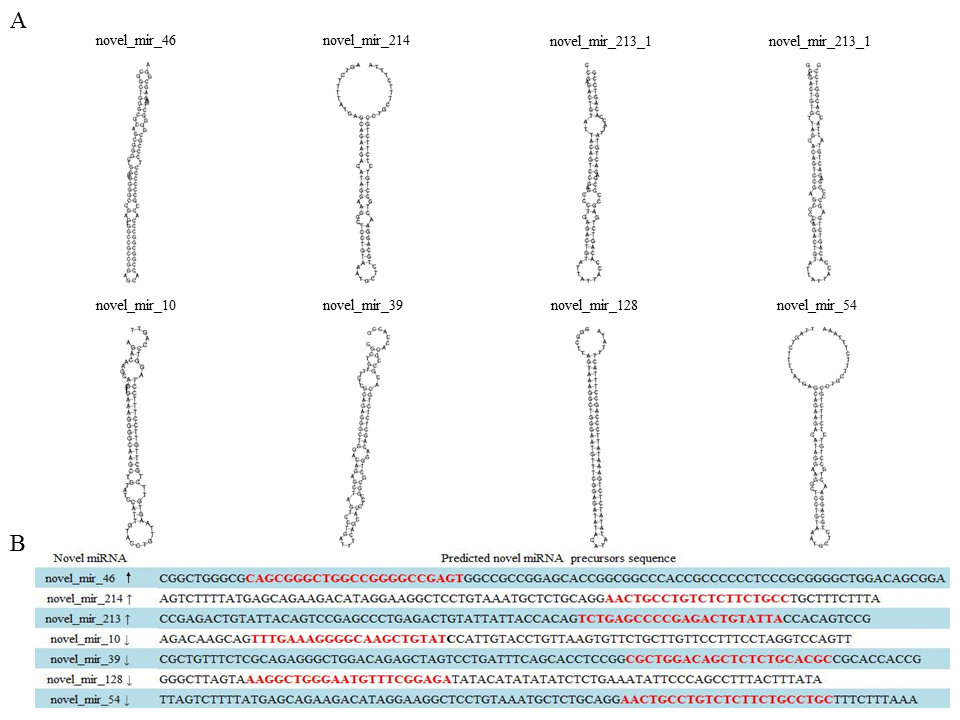


**Figure 3**
