# Supplementary figures and images for: Impaired GABA synthesis, uptake and release are associated with depression-like behaviors induced by chronic mild stress
Source: Transl Psychiatry. 2016 Oct 4;6(10):e910–. doi: 10.1038/tp.2016.181 (PMC5315548; doi:10.1038/tp.2016.181)

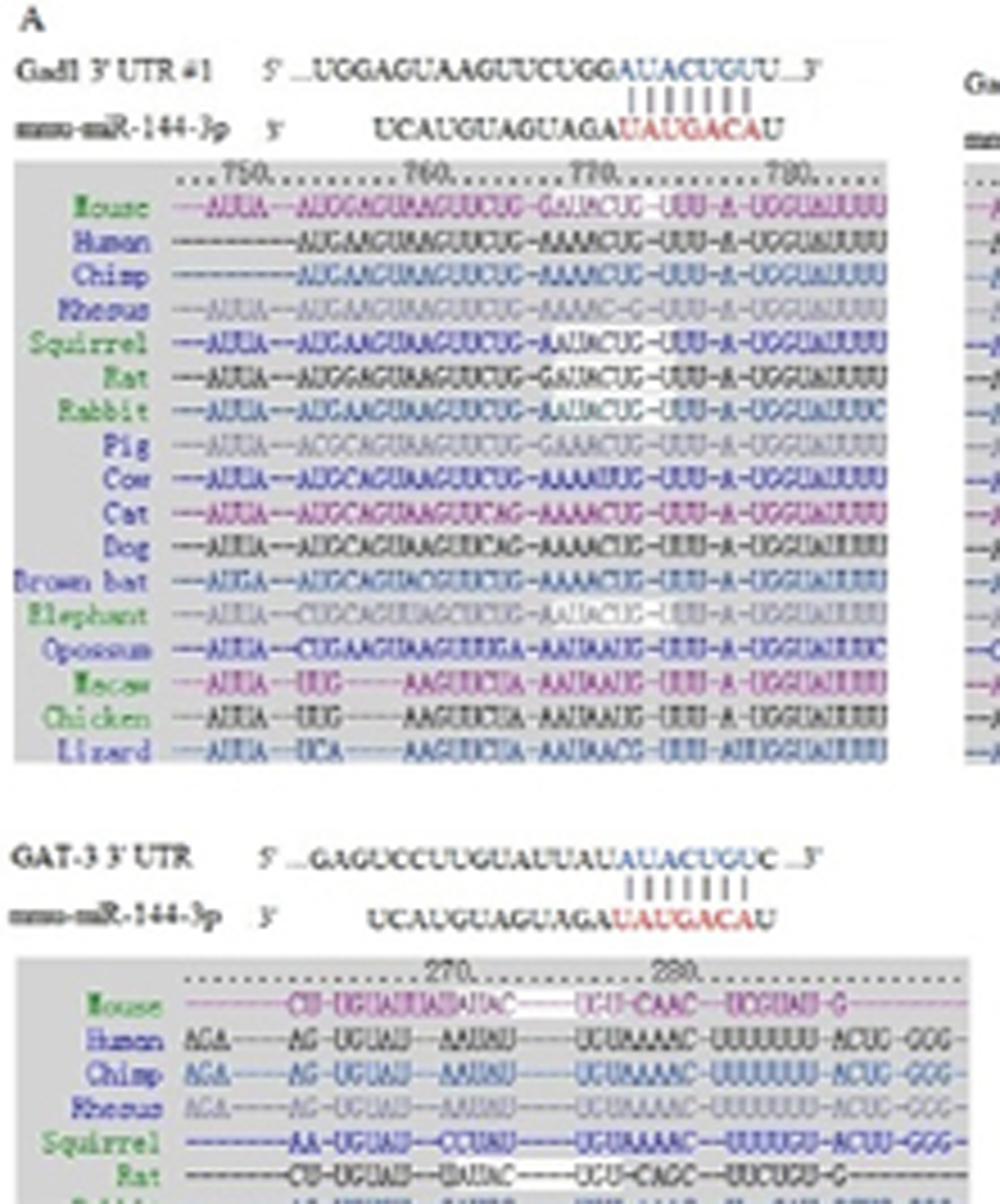

Supplement: Supplementary Figure 1 [file tp2016181x2.tif]

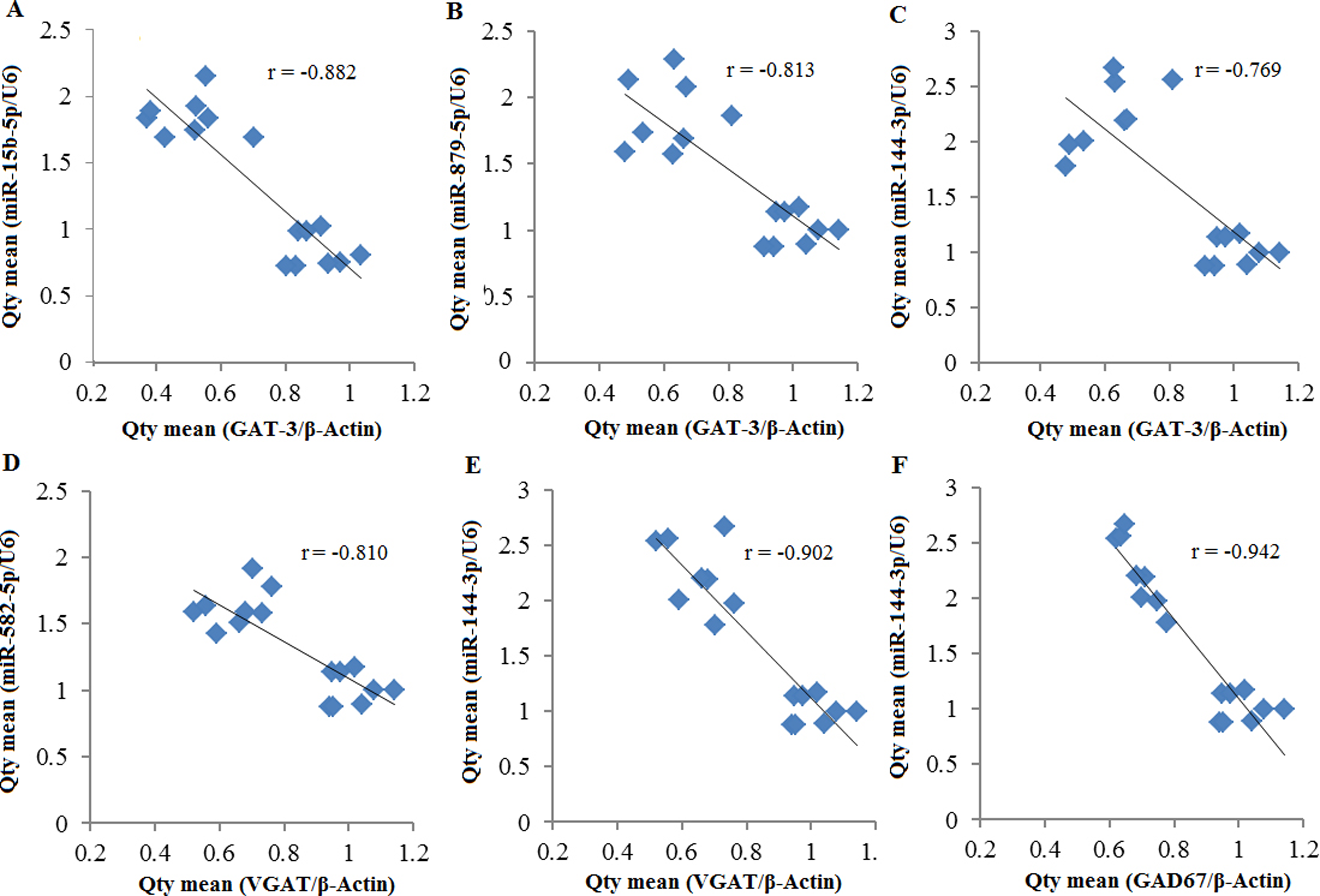

Supplement: Supplementary Figure 2 [file tp2016181x3.tif]

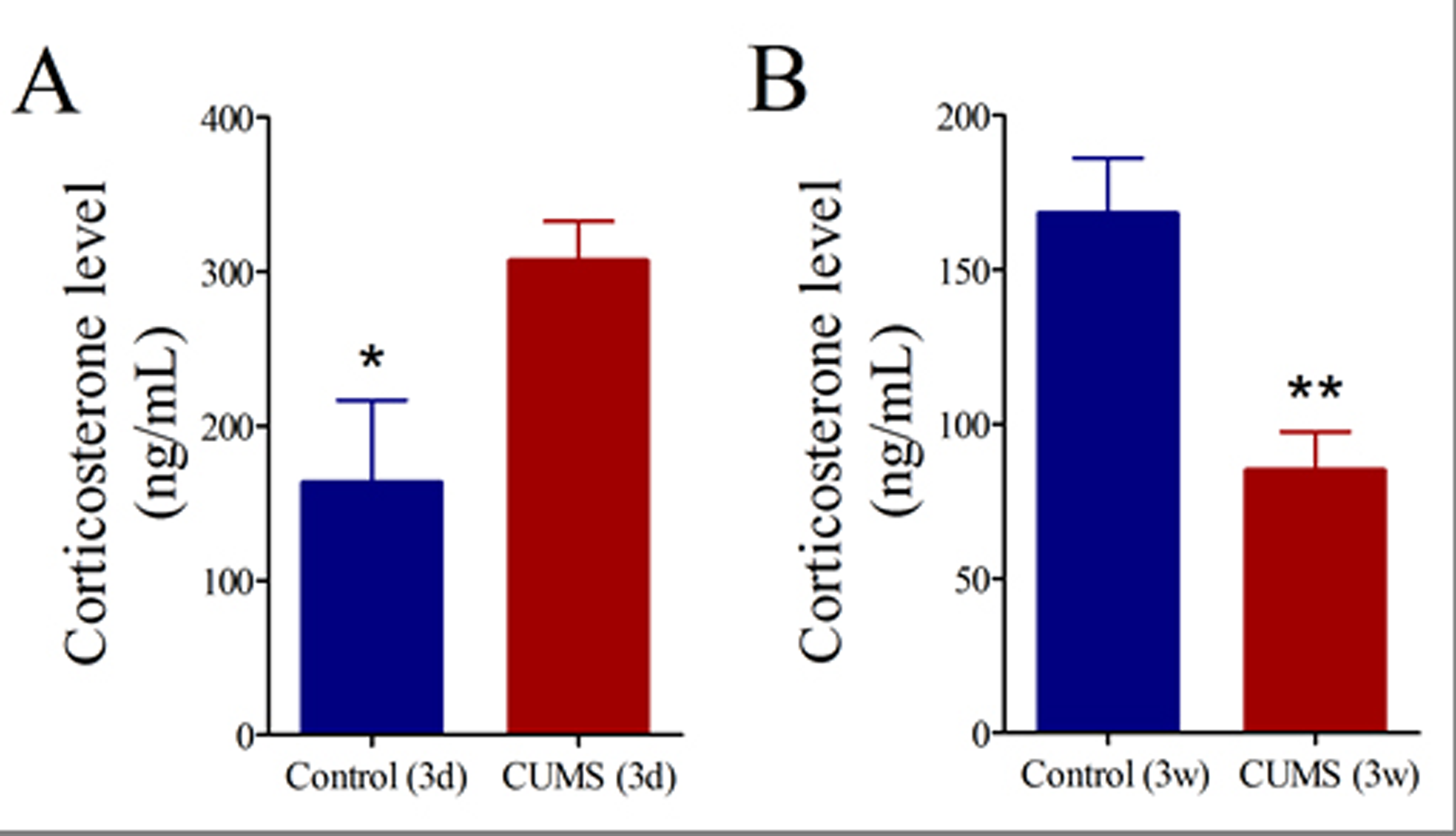

Supplement: Supplementary Figure 3 [file tp2016181x4.tif]
