# Supplementary material for: Impaired GABA synthesis, uptake and release are associated with depression-like behaviors induced by chronic mild stress
Source: Transl Psychiatry. 2016 Oct 4;6(10):e910–. doi: 10.1038/tp.2016.181 (PMC5315548; doi:10.1038/tp.2016.181)
Supplement: Supplementary Legends [file tp2016181x8.docx]

**Legends for Supplemental Figures and Tables**

**Supplementary Figure 1:** The conservation of selected miRNAs with their predicted target mRNA in terms of binding sequences across different vertebrate species. **A)** shows the conservation of miR-144-3p with their predicted target *GAD1* across different vertebrate species; **B)** shows the conservation of miR-144-3p with their predicted target *VGAT* across different vertebrate species; **C)** shows the conservation of miR-144-3p and miR-15b-5p with their predicted target *GAT-3* across different vertebrate species.

**Supplementary Figure 2:** Correlation between miRNAs and its prediction target expression in PFC tissue from subjects with depression-like mice and controls. **A)** shows the correlation between GAT-3 and miR-15b-5p (r=-0.882; *p*<0.001). **B)** shows the correlation between GAT-3 and miR-879-5p (r=-0.813; *p<*0.001). **C)** shows the correlation between GAT-3 and miR-144-3p (r = -0.769; *p*<0.001). **D)** shows the correlation between VGAT and miR-582-5p (r = -0.810; *p*<0.001). **E)** shows the correlation between VGAT and miR-144-3p (r=-0.902; *p*<0.001). **F)** shows the correlation between GAD67 and miR-144-3p (r=-0.942; *p*<0.001). Data are the mean ± SEM. qPCR of miRNAs and mRNAs were analyzed in 8 mice per group.

**Supplementary Figure 3:** The levels of serum corticosterone in the depression-like mice induced by chronic unpredicted mild stress (CUMS). The level of corticosterone was measured by ELISA. **A)** shows the levels of serum corticosterone in CUMS-treated mice (red bar, n=10) and control mice (blue bar, n=5, *p*<0.05) for three days. Corticosterone rises in the early three days of CUMS treatment. **B)** shows the levels of serum corticosterone in CUMS-treated mice (red bar, n=8) and control mice (blue bar, n=6, *p*<0.01) for three weeks. Corticosterone decreases after CUMS treatment for three weeks.

**Supplementary Table 1:** qRT-PCR prime information.

**Supplementary Table 2:** 3′-untranslated region (UTR) and site-directed mutation prime sequence GAD1, VGAT and GAT-3.

**Supplementary Table 3:** The prediction scores of selected miRNAs and their targets (Data from targetscan-http://www.targetscan.org/).
